# Flexible encoding of multiple task dimensions in human cerebral cortex

**DOI:** 10.3389/fcogn.2024.1438390

**Published:** 2024-07-24

**Authors:** Benjamin J. Tamber-Rosenau, Allen T. Newton, René Marois

**Affiliations:** ^1^Department of Psychology, Vanderbilt University, Nashville, TN, United States; ^2^Vanderbilt Vision Research Center, Vanderbilt University, Nashville, TN, United States; ^3^Department of Psychology, University of Houston, Houston, TX, United States; ^4^Vanderbilt University Institute of Imaging Science, Vanderbilt University Medical Center, Nashville, TN, United States; ^5^Department of Radiology and Radiological Sciences, Vanderbilt University Medical Center, Nashville, TN, United States

**Keywords:** domain-general, cognitive resources, pattern analysis, MVPA, decoding, fMRI, multiple demand network, task positive network

## Abstract

**Introduction:**

Cognitive models have proposed that behavioral tasks can be categorized along at least three dimensions: the sensory-motor modality of the information, its representational format (e.g., location vs. identity), and the cognitive processes that transform it (e.g., response selection). Moreover, we can quickly and flexibly encode, represent, or manipulate information along any of these dimensions. How is this flexibility in encoding such information implemented in the cerebral cortex?

**Methods:**

To address this question, we devised a series of functional magnetic resonance imaging (fMRI) experiments in each of which participants performed two distinct tasks that differed along one of the three dimensions.

**Results:**

Using multivariate pattern analysis of the fMRI data, we were able to decode between tasks along at least one task dimension within each of the cortical regions activated by these tasks. Moreover, the multiple demand network, a system of brain regions previously associated with flexible task encoding, was largely composed of closely juxtaposed sets of voxels that were specialized along each of the three tested task dimensions.

**Discussion:**

These results suggest that flexible task encoding is primarily achieved by the juxtaposition of specialized representations processing each task dimension in the multiple demand network.

## Introduction

Human behavior is flexible: memorized information and arbitrary rules are combined with inputs from any sensory modality to yield behaviorally adaptive responses in any motor modality. This cognitive flexibility reflects a combination of highly specified peripheral sensory and representational systems with more flexible central systems in frontal and parietal association cortex that integrate information across representations (e.g., Bunge et al., 2000; Nystrom et al., 2000; Jiang and Kanwisher, 2003; Koechlin et al., 2003; Badre and Wagner, 2004; Badre, 2008; Niendam et al., 2012; Power and Petersen, 2013; Tamber-Rosenau and Marois, 2016; Noyce et al., 2017). Flexible encoding of a wide array of computations (Woolgar et al., 2011, 2016; Fedorenko et al., 2013; Erez and Duncan, 2015; Cole et al., 2016; Etzel et al., 2016; Shashidhara et al., 2020; Shashidhara and Erez, 2021) has been specifically attributed to a fronto-parietal task positive or multiple demand (MD) network (Duncan and Owen, 2000; Fox et al., 2005; Duncan, 2010; Assem et al., 2020, 2022, 2024), surrounded by neighboring brain regions characterized by additional, weaker shared activity (Assem et al., 2020). Generally, MD regions perform similarly across tasks (Woolgar et al., 2011; Erez and Duncan, 2015; Assem et al., 2024), fractionating into only two sub-networks operating over distinct timescales (Dosenbach et al., 2008; Crittenden et al., 2016). Correspondingly, multivariate pattern analysis (MVPA) research has revealed flexible encoding of stimulus *category* (e.g., faces vs. houses), response *identity* (e.g., finger used for manual response) as well as task and stimulus-response mapping rules in MD regions (Woolgar et al., 2016; Assem et al., 2020, 2022, 2024). However, no single study has explored the systematic representation of possible task dimensions not only in the MD network but also throughout the cerebral cortex. To what extent are task dimensions flexibly represented in the MD network and the rest of the cerebral cortex, and is this flexibility a result of a neural process that is truly functionally pluripotent, devoid of any encoding preference or bias for any task dimensions? Such a pluripotent process may not only be implied by the notion of an MD network, but also by frequently invoked concepts in the cognitive psychology literature such as domain-general resources, central (or universal) bottlenecks, attentional resources, or a general fluid intelligence factor, making it critical to understand if pluripotency is a real property of systems within the human brain.

To systematically assess flexible task information encoding, we guided our experimental approach with inspiration from Wickens (1980, 1984, 2002, 2008)'s multiple resources model of cognitive processing. Modifying Wickens's scheme to broaden its applicability, tasks can be considered to vary along at least three dimensions: sensorimotor *modality*, information representational *format*, and cognitive *process*. Akin to the factors in a factorial design, each task dimension has multiple discrete levels (e.g., spatial, object, and verbal categories; c.f., Nee et al., 2013). The dimensions together define a multidimensional task matrix. Tasks are characterized by the cell(s) they occupy in this matrix: for example, a visual-manual sensorimotor *modality* task relying on the spatial representational *format* and requiring the response selection cognitive *process*.

In the present study, we used MVPA of fMRI data to compare pairs of tasks that required distinct levels of each task dimension, as MVPA allows identification of brain regions whose activation patterns distinguish between task dimension categories, i.e., would contain spatially segregated neural ensembles specialized for distinct modalities, formats, or processes. Our analytical approach hinges critically on what aspects of neural activity can drive MVPA classification. Specifically, MVPA assesses the “information content” (c.f., Kriegeskorte et al., 2006) of an entire ROI. When overall differences in activation amplitude across conditions is subtracted out in MVPA applied to a given ROI (as we did in the present research; see Materials & Methods, Esterman et al., 2009; Tamber-Rosenau et al., 2011), the results of the MVPA is driven exclusively—for each participant—by differences in the spatial distribution of activity within the ROI, thus revealing whether any given brain region contains neural ensembles that distinctly code each task condition. Such fine-scale spatial segregation can be quite variable from person to person (c.f., Fedorenko et al., 2010, 2012), making a tool that can detect segregation patterns that are idiosyncratic to individual participants, such as MVPA, particularly useful (Peelen and Downing, 2007; also see Peelen et al., 2006; Tamber-Rosenau et al., 2013; Lee and McCarthy, 2014).

The crux of our study does not simply address the question of whether the human brain shows domain generality—after all, the impressive flexibility of our cognitive capacities demonstrates as much—but more specifically, we investigate at what level of organization such domain generality may arise. At one extreme, domain-generality may be a brain- or cortex-wide property, whereby any given individual brain region shows domain specificity. At the other extreme, domain-generality could be reflected in individual nerve cells or neural ensembles if those are truly pluripotent and do not preferentially encode any one of the task dimensions. It is also conceivable that domain generality is an emergent property of a brain region, whose encoding flexibility stems from the region containing multiple neural ensembles each specialized for different dimensions. According to the latter possibility, the entire ROI would exhibit domain-generality by virtue of containing distinct neural ensembles each functionally specialized for any given task dimension. While it is clear that the cerebral cortex as a whole shows at least some functional specificity (e.g., consider the distinct functional properties of the primary visual and auditory cortex), it is less clear whether brain regions in association cortex show ROI-level or neuronal-level domain generality. Thus, along with the novelty stemming from considering process, format, and modality task dimensions in a single overarching framework, our study is designed to distinguish between these different scales of domain generality, which MVPA is well suited to tease apart. In our view, while the identification of domain-general, multiple demand, or task positive brain networks in the support of human cognitive flexibility has been a major step forward in our understanding of the neural basis of human cognitive flexibility, this understanding is still incomplete. It is also critical to consider how flexibility or domain-generality in any given network or brain region is achieved—whether by true pluripotency similar to the notion of a domain-general cognitive resource from psychology, or instead by the colocalization of multiple specialized resources or neural structures.

To address these issues, here we took a two-pronged approach. First, we used an ROI-based analysis to identify brain regions that were activated by both tasks varying along a dimension and then used MVPA to test for distinct voxel-based biases or neural ensembles supporting the two tasks. Second, using an exploratory “searchlight” analysis (Kriegeskorte et al., 2006), we searched for brain regions that decoded along each of the three task dimensions. Brain regions that are functionally invariant to a particular task dimension should exhibit no MVPA decoding between task levels along that dimension, meaning that they should not contain segregated neural populations supporting performance of tasks varying along the manipulated dimension. A completely domain-general, pluripotent brain region then would be invariant to all three task dimensions^1^ (i.e., it would not exhibit functional bias along any one of the three task dimensions).

**Figure 1:**
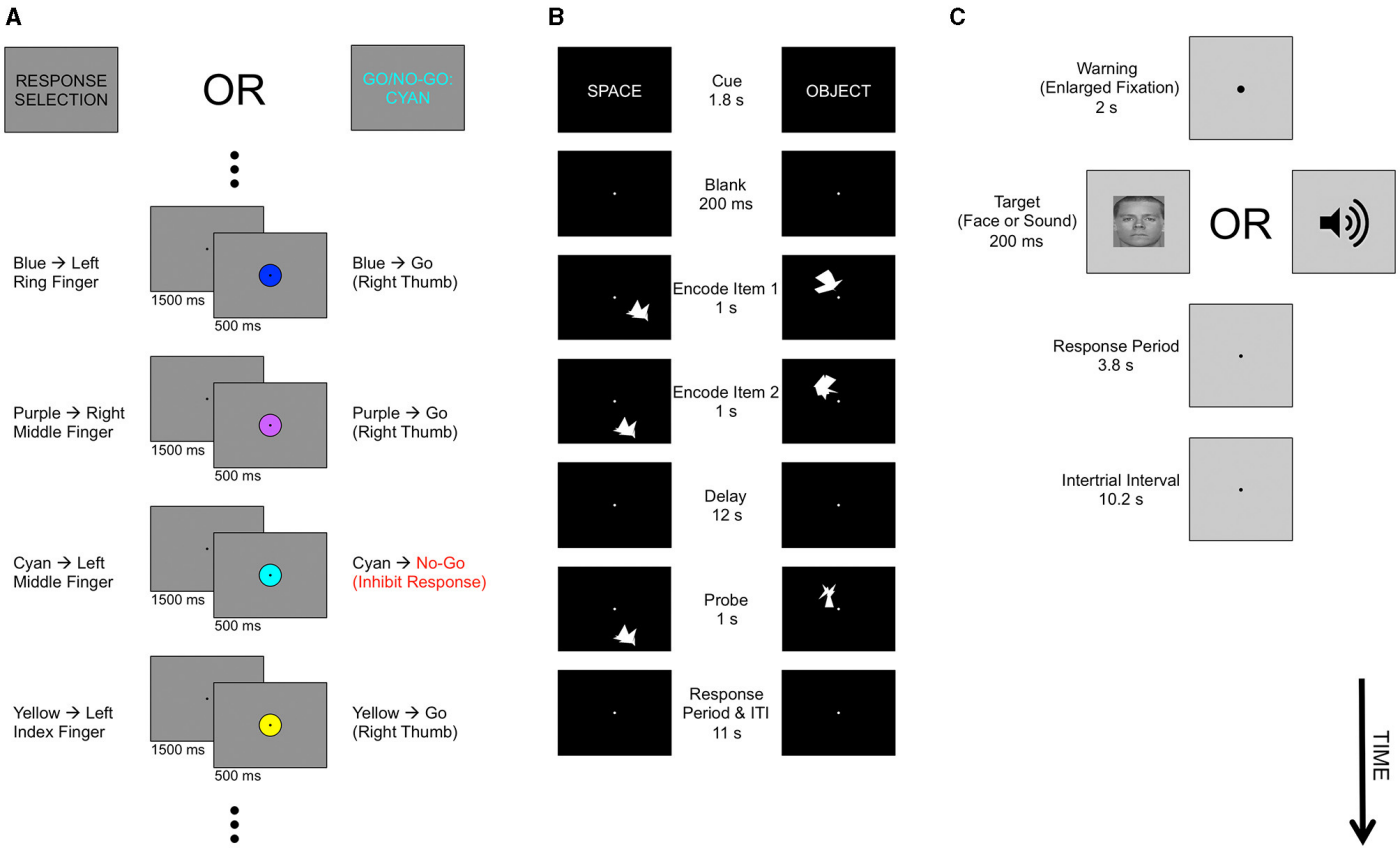
Tasks. **(A)** Cognitive process. Each run of the Process experiment contained two tasks, the Response Selection (RS) task and the Go/No-Go (GNG) task. Task identity was cued at the beginning of each 24-trial block via the phrases “Response Selection,” or “Go/No-Go,” presented visually. The visual stimuli were identical for the two tasks (center column), but the appropriate response to each stimulus varied by task (left column: RS; right column: GNG). On each trial, a central disk was presented in one of six colors. Disk presentation alternated with fixation periods. In the RS task, each color instructed the participant to press a distinct button. In the Go/No-Go task, five “Go” colors instructed the participant to press a single button. A sixth, “No-Go,” color instructed the participant to withhold any motor response to that trial, i.e., to inhibit the prepotent “Go” response. **(B)** Representational format. Each run of the Format experiment contained two tasks, the Object Spatial Location task and the Object Shape task. At the beginning of each trial, we presented a visual cue (the words “Space” or “Object” for the Location and Shape tasks, respectively) followed by the sequential presentation of two sample items (the encoding period). Participants memorized the cued dimension of the samples, and maintained them in memory over a 12 s delay. In the Location task, subjects maintained the spatial location of each sample item. In the Shape task, subjects maintained the shape of each sample item. Sample items in both tasks were complex 16-sided polygons. At the end of the delay period, we presented a single probe item and subjects indicated via button press if the probe was a match to one of the sample items on the cued dimension, or a non-match (i.e., was different from both sample items). **(C)** Sensorimotor modality. On each trial of the Modality experiment, we presented a warning cue (enlarged fixation) followed by a visual (face) or auditory stimulus. Participants made a speeded arbitrary stimulus-response mapping with 3 possible face-button mappings and three possible sound-speech mappings (see Materials and Methods). Task screens are not drawn to scale.

The present study (Figure 1) investigated tasks varying in Process by comparing a response selection (RS) task and a response inhibition (RI) task. For the Format experiment, we compared object location and object shape working memory (WM) encoding. Finally, for the Modality experiment, we reanalyzed visual-manual (VM) and auditory-vocal (AV) stimulus-response mapping tasks (Tombu et al., 2011; Tamber-Rosenau et al., 2013).

## Materials and methods

### Overview

In each experiment, subjects performed two distinct tasks that loaded primarily on distinct processes (RS or RI), formats (location or shape), or modalities (visual-manual or auditory-vocal). In the Process and Format experiments, we defined ROIs based on the univariate overlap of activation for the two tasks within each experiment using standard general linear model (GLM) analyses and open contrasts of each task vs. un-modeled baseline (these liberal contrasts were employed to avoid discarding brain regions that may exhibit task-invariant decoding) (Dux et al., 2006). We subjected each ROI to MVPA to determine if it contained neural ensembles or sub-regions that were specialized within the tested dimension. A similar analysis of the Modality experiment is reported in Tamber-Rosenau et al. (2013) and here extended to the Process and Format ROIs. All MVPA was carried out using support vector machine learning algorithms in LibSVM (Chang and Lin, 2011) and standard leave-one-run-out procedures to ensure independence of training and test data, accounting for mean activation via mean-subtraction. MVPA classification rates in each ROI were computed at a series of timepoints locked to the start of each trial to obtain MVPA decoding timecourses, and we report peak classification for each ROI including *t-*tests vs. chance (50% correct classification).

We also subjected data from each experiment to “searchlight” MVPA (Kriegeskorte et al., 2006) in which we collapsed trials over time by fitting a GLM to each run and performed MVPA on parameter (beta) weights from these GLMs. The searchlight MVPA analysis is unlikely to be influenced by motion or other nuisance factors, including response time (at the level of single trials), because we regressed out such variables prior to searchlight MVPA. Searchlight results were projected onto an inflated brain surface based on a Talairach-transformed COLIN27 brain (Holmes et al., 1998) and thresholded using the false discovery rate procedure to correct for multiple comparisons (Benjamini and Hochberg, 1995).

### Participants

All experiments were carried out in accordance with a protocol approved by the Vanderbilt University Institutional Review Board. Each participant gave informed consent and passed a standard safety screening.

The same set of 16 participants (8 males, 8 females, mean age of 23.9 years, range 20–30 years) participated in both of the new experiments (Process and Format). An additional two subjects participated in only one of the new fMRI experiments and thus were excluded from all analyses. Data for the Modality experiment were drawn from a previous publication (Tombu et al., 2011) and included 12 subjects (7 males, 5 females, 21–33 years of age). Our data were drawn from two distinct sets of subjects and collected on two scanners with different field strengths. However, all statistical tests were performed within-experiment and the only cross-experiment analyses examined overlap of activation or decoding across experiments, making the use of distinct subject sets and scanners unlikely to drive our results.

### Cognitive process experiment: experimental design and statistical analysis

#### Behavioral training

All participants performed an initial behavioral training session in which they practiced the Process tasks (Figure 1A). During this session (approximately 30 min, excluding consent and safety screening), each participant memorized the color-to-finger response mapping for the Response Selection (RS) task and became familiar with both the RS and response inhibition (RI) Go/No-Go (GNG) tasks.

#### Tasks

Tasks in this and all other experiments were displayed using Matlab with the Psychophysics Toolbox (Kleiner et al., 2007) and custom code. The Process experiment contained two tasks (Figure 1A): the RS task and the RI (GNG) task. Each of these tasks was designed to load heavily on a distinct, widely-invoked executive process—response selection for the RS task and response inhibition for the RI task (Donders, 1868/1969; Welford, 1952; Logan et al., 1984; Pashler, 1984, 1994; Logan, 1985; Miyake et al., 2000; Aron, 2007)—while loading little or not at all on the opposing process. The RS task consisted of an arbitrary six-alternative visual-manual stimulus-response mapping. Each trial consisted of the presentation of a colored disk at fixation (radius: 2.21 degrees of visual angle) for 500 ms, followed by a 1,500 ms fixation period until the beginning of the next trial. Subjects indicated the color of the presented stimulus (blue, cyan, yellow, peach, purple, and brown) by pressing one of six buttons using the index, middle, or ring finger of each hand. Color-to-finger mappings were memorized prior to scanning (see Behavioral Training below). Each of the six colors was presented four times per block of 24 trials; these blocks alternated with same-length blocks of RI trials.

The RI task was identical in visual and timing parameters to the RS task. However, instead of each colored disk being associated with a specific manual response, subjects performed a GNG decision on each trial: 5 of the colors were associated with the same Go response (right thumb) and one color was associated with the withholding of all responses—a No-Go trial. The No-Go color was varied from scanning run to scanning run such that each of the six colors functioned as the No-Go color in one or two runs of the Process experiment.

For both the RS and GNG tasks, the order of color presentation was pseudo-randomized subject to the constraints that all colors appeared an equal number of times in each block, and that the No-Go color for that imaging run never appeared in the first 4 trials of a block of either task. This latter constraint was applied to the GNG task to allow time for prepotency of “Go” responses to build at the start of each block. The same constraint was also applied to the RS task to ensure that all stimulus visual attributes and timing parameters were identical between GNG and RS tasks.

The task was cued prior to each block by visual presentation of the words “RESPONSE SELECTION” for the RS task, or of “GO/NO-GO:” to the left of the name of the No-Go color (e.g., “GO/NO-GO: RED”) for the RI task. The block task cue was presented for 4 s and was followed by a 500 ms fixation period before block onset. The RS block cues were always presented in black, and the GNG block cues were always presented in the No-Go color. In addition, participants were instructed as to the No-Go color at the beginning of each fMRI run. All stimuli in both tasks were presented on a medium gray background, with a black fixation point in the center of the screen at all times except during block cue presentation. Each task run consisted of alternating GNG and RS blocks of 24 trials each, with 6 s cue periods before each block (during which fixation was presented for 1.5 s, the cue was presented for 4 s, and the remaining time consisted of a fixation point), and with the task for the first block of a run alternating from run to run. Though the RS and GNG tasks were blocked, we took advantage of the fact that the timings of the No-Go color trial in the GNG condition and of the corresponding color trial in the RS condition were pseudo-randomized within their respective blocks to resolve event-related activation to these trial types (Donaldson et al., 2001; Braver et al., 2003). We thus used these event-related activations for both univariate and multivariate analyses of the Process experiment.

#### fMRI procedure

The Process experiment was carried out at the Vanderbilt University Institute of Imaging Science using a Philips 7 Tesla fMRI scanner and a 32-channel SENSE (parallel imaging) head coil. For each subject, the process scanning session included a 1 mm isotropic resolution T1-weighted structural scan (174 1-mm sagittal slices of 224 × 224 voxels, FOV 224 × 224 mm, TR 4.3 ms, TE 1.9 ms, flip angle 7 degrees, SENSE factor 3) and a series of 3 mm isotropic resolution echo-planar imaging functional scans (35 3-mm axial slices of 80 × 80 voxels, FOV 240 × 240 mm, no gap, TR 2.0 s, TE 25 ms, flip angle 65 degrees, SENSE factor 3). Process experiment sessions included 13.125 ± 1.025 runs (range 11–14), each containing 171 volumes (duration 342 s). Each functional scan was preceded by 10 dummy volumes. We chose to acquire standard-resolution (3 mm isotropic voxels, 2 s TR) images at 7 Tesla in order to maximize signal strength (see, e.g., Ogawa et al., 1993; Gati et al., 1997) rather than spatial resolution.

#### fMRI preprocessing

All fMRI data were preprocessed using BrainVoyager QX or using custom Matlab (MathWorks, Natick, Massachusetts, USA) code except as noted below. Data were registered to the structural MRI using standard procedures. Following registration, data were corrected for slice acquisition time, corrected for motion, subjected to linear trend removal and a temporal high-pass filter (3 cycles per run) to remove slow drift components in the signal, and transformed to Talairach and Tournoux (1988) template space.

Because preliminary analyses showed that spatial position estimate outputs from BrainVoyager's motion correction could support above-chance decoding of task using MVPA, we turned to ANTs software (Avants et al., 2011) for diffeomorphic motion correction that does not assume rigid body motion. Non-rigid motion correction may be justified at 7 Tesla due to increased image distortions and field inhomogeneity compared to 3 Tesla fMRI. ANTs motion correction was performed on the ACCRE supercomputing cluster at Vanderbilt University and removed nearly all detectable motion from the data. Though this procedure eliminated a possible confound from our data, preliminary analyses using BrainVoyager's standard motion correction algorithms yielded broadly similar results to our final results, demonstrating that our findings cannot be explained by the use of diffeomorphic motion correction.

Next, we removed nuisance components from the data using a general linear model (GLM). Specifically, we used the NeuroElf (Jochen Weber, Columbia University). Matlab toolbox functions for interacting with BrainVoyager proprietary file formats, along with custom Matlab code, to extract the z-transformed residuals from a GLM that included only regressors for task block type (convolved with a canonical HRF) and z-transformed motion estimates from the initial BrainVoyager motion correction. These residuals were then added to a constant (to make all values positive, a requirement of the proprietary BrainVoyager VTC file format) and used for subsequent analyses. Thus, block-level effects and motion or position differences across blocks were removed from our data prior to the main analyses, allowing us to conduct all analyses based on event-related signals as detailed above.

Finally, we masked all data using a liberal anatomical mask. Functional (3 mm) voxels were included in the mask if they contained at least one anatomical (1 mm) voxel that was identified as brain tissue in at least 8 of 16 subjects using BrainVoyager's automatic tissue segmentation function (or manual segmentation when the automatic function failed to remove the skull).

#### Univariate fMRI analysis and functional region definition

We analyzed the Process experiment using a conventional group GLM (Friston et al., 1995) voxel-wise analysis, with subject treated as a random effect, in which we estimated parameter weights for a series of regressors convolved with a canonical hemodynamic response function (Boynton et al., 1996). The GLM included both block-level regressors to account for variance explained by the block structure of the experiment, and event-related regressors to account for variance explained by punctate events (individual trials). The set of event-related regressors included cue presentation epochs (separate regressors for each task), Go trials (boxcar with duration equal to the trial-level response time), No-Go trials (boxcar with duration equivalent to the mean Go response time during that run), Response Selection trials of the No-Go color (RS_NGC; boxcar with duration equal to the trial-level response time), and Response Selection trials of any other color (RS_Other; boxcar with duration equal to the trial-level response time). We also included regressors for error trials, separately for the GNG and RS tasks. We used regressor boxcars with duration equal to response time in order to account for time-on-task effects (Grinband et al., 2008). Though we estimated No-Go “response times” by using mean Go response times, likely representing an upper bound on No-Go processing times (Logan and Cowan, 1984; Verbruggen and Logan, 2008; Matzke et al., 2013; Logan et al., 2014), minor variations in regressor boxcar duration do not negatively impact MVPA (Woolgar et al., 2014), the main analysis of interest in this study.

We identified ROIs for later multivariate analysis via contrasts between parameter weights from the univariate GLM. Specifically, we used the conjunction of the open contrasts of the No-Go and the RS_NGC regressors. Prior to taking the conjunction of the open contrasts, each open contrast map was separately smoothed with a Gaussian kernel (FWHM = 3 mm) to reduce any effects of noise and to account for minor variations in spatial alignment. Note that these open contrasts are liberal in that they are sensitive to both task-specific demands and more general demands (e.g., neural response to the presentation of a visual item regardless of task). This choice of a liberal criterion to identify ROIs is with an eye toward isolating all brain regions potentially engaged by both tasks, and has previously been used successfully to examine modality-sensitive and modality-invariant brain regions (Ivanoff et al., 2009; Tamber-Rosenau et al., 2013).

GLM conjunction contrasts were thresholded at a voxelwise alpha of 0.05 and then corrected for multiple comparisons using the BrainVoyager Cluster-based Statistical Threshold Estimator plug-in to achieve a familywise error rate of 0.05, yielding an extent threshold of 42 functional voxels. The resulting contrast maps were used to identify regions of interest: local maximum contrast value voxels were identified in each activated brain region without constraints. Once the local maxima were identified, we created spherical ROIs (radius: 9 mm, including a total of 123 functional voxels) centered on these voxels. Following this procedure, in order to remove highly redundant ROIs, we culled ROIs that overlapped substantially with one another. The final set of Process ROIs is described in Supplementary Table S1.

#### ROI-based multivariate pattern analysis

In each set of ROIs—the Process ROIs described above, the Format and Modality ROIs (see below), and in a set of MD network ROIs based on previously published MD coordinates (Duncan, 2010) (see Supplementary Table S4)—we performed MVPA to test for sensitivity to task identity. Specifically, we compared RS_NGC trials to No-Go trials, in effect testing whether neural activity in each ROI was sensitive to which process (RS vs. RI, respectively) was demanded in otherwise matched trials.

All MVPA was carried out using LibSVM (Chang and Lin, 2011) and custom Matlab code. Support vector machine classifiers were trained and tested using a leave-one-run-out approach. The cost parameter was fixed at 1 as is standard practice for SVMs in fMRI analysis. Classifiers were trained and tested on patterns of activity at single points in time ranging from −6 to 16 s relative to trial onset. Results were aggregated over leave-out folds, averaged across subjects, and concatenated in time to yield event-related MVPA timecourses, conceptually similar to conventional event-related averages of BOLD signal. Such timecourses provide a built-in control to ensure that sensitivity to task is not merely the result of holding different task sets or other preparatory states across the blocks of our experiments, as signal prior to trial onset should reflect only this preparatory activity. To the extent that pattern classification during the three sampled fMRI volumes prior to trial onset (here, −6 to −2 s) is not significantly different from chance, we need not be concerned with task block-level effects contaminating our analysis.

Statistical significance of event-related MVPA timecourses was evaluated at each timepoint using one-tailed *t*-tests vs. chance performance (50%). We used one-tailed tests because our goal was to identify regions with above-chance decoding, as there is no clear interpretation of significantly below-chance decoding. All tests of decoding performance were corrected for multiple comparisons via Bonferroni correction for the number of regions of interest within each ROI set, i.e., Process, Format, Modality, or MD ROIs. In each ROI, we extracted peak decoding performance in the fMRI volumes 2–10 s after trial onset, which included the expected peak response after considering hemodynamic lag.

#### Searchlight multivariate pattern analysis

In addition to the ROI-based pattern analyses described above, we performed “searchlight” multivariate pattern analyses over the entire (masked) brain volume (Kriegeskorte et al., 2006). Each functional voxel was treated as the center of a new searchlight locale (radius = 6 mm, including 33 functional voxels). MVPA procedures were identical to those described above for ROI-based MVPA except that instead of calculating event-related timecourses, we used a time-compression procedure to capitalize on signal from the entire trial or task phase. Specifically, each individual task run was subjected to a fixed-effects, single-run GLM using regressors identical to those in the main GLM analysis described above. Parameter weights for conditions of interest (RS_GNG vs. No-Go) were extracted for each voxel in each locale. These run-based parameter weights were then used as training and testing data for MVPA using a leave-one-run-out approach, as above. MVPA classification results were then projected into the three-dimensional brain volume for each subject, averaging across leave-one-run-out folds, to yield an MVPA performance map. These maps were tested against chance using paired *t*-tests at each voxel to yield group maps in which subject was treated as a random effect, comparable to the statistical maps output by standard group GLM analyses. All such maps were masked with the anatomical brain mask used for the univariate analyses prior to being projected onto a Talairach-transformed, inflated brain surface model derived from the COLIN27 brain (Holmes et al., 1998) using BrainVoyager.

### Representational format experiment: experimental design and statistical analysis

#### Behavioral training

All participants performed an initial behavioral training session (lasting approximately 30 min) in which they practiced both tasks of the Format experiment.

#### Task

The Format experiment contained two tasks (Figure 1B): the Object Spatial Location task and the Object Shape task. In both tasks, participants performed a series of working memory trials. A white fixation point was presented on a black screen throughout the experiment except when textual cues were provided at fixation. Each trial began with a 1.8 s cue indicating the upcoming task, i.e., the word “SPACE” (Location task) or “OBJECT” (Shape task) displayed in white. After a 200 ms fixation screen, two sample items were sequentially presented for and encoding period of 1 s per item. The encoding period was followed by a 12 s fixation-only delay, which was followed by presentation of a probe item for 1 s and then a 3 s response interval. Participants indicated if the probe matched either of the sample items (“Match” response, right index finger) or did not match either item (“Nonmatch” response, right middle finger). Each trial was followed by an 8 s inter-trial interval (ITI; fixation only), yielding a total of 11 s from the offset of the probe until the task cue that began the next trial. We designed the Format experiment to use long ITIs so that we could to resolve event-related activations for both univariate and multivariate analyses. Trials were blocked by task, though as stated above we also cued the task immediately before each trial to reduce memory requirements because of the long ITI and delay period durations. Importantly, the stimuli on each trial imposed demands on only one of the formats. Specifically, in each trial of the Location task, only the spatial location of stimuli was varied between items while item shape was held constant for all items. In each trial of the Shape task, only the item shape was varied between items while item location was held constant for all items. By varying only the task-relevant dimension on each trial, we made the task-irrelevant dimension uninformative for response choice. This scheme reduced the chance that participants would become confused about which task to perform and would attempt to encode both task dimensions instead of performing only the cued task. Moreover, by using both bottom-up (i.e., presence of stimulus variation in the task-relevant dimension only) and top-down (task instructions) cues for distinguishing between Location and Shape trials, we increased the distinctiveness of the Format tasks and hence the power of MVPA in distinguishing between neural ensembles involved in each of these tasks.

In each trial of both the Shape and Location tasks, we generated three distinct stimuli. Two of these were randomly chosen as sample items. On 50% of trials, the remaining item was used as the probe (Non-match trials), while on the other (Match) trials, the probe was identical to one of the sample items and we discarded the third generated item. All stimulus and probe items consisted of novel, randomly generated, irregular polygons positioned within the viewable area of the projection screen in the 7 Tesla scanner (approximate dimensions, 21.6 degrees of visual angle wide × 8.4 degrees of visual angle high). Stimuli were generated by drawing a 16-sided polygon whose vertices were separated by 22.5 degrees of rotational angle and were positioned at a random radius in the range 0.66–1.33 degrees of visual angle, subjected to jitters of 11.25 degrees of rotational angle, and then distorted further. These final distortions were implemented by moving each point in the polygon a set distance, controlled by the *distortion parameter* (which was in turn controlled by the staircasing; see Staircasing below), in a random direction. Item locations were generated as follows: A random point within the viewable screen area was chosen as the center of the original (non-distorted) polygon. A second stimulus location (on Location trials) was chosen by moving a set distance, controlled by the *distance parameter* (also controlled by the staircasing; see Staircasing), in a random direction, subject to the constraint that the chosen location had to be within the viewable area of the display. A third stimulus location (to be used for non-match trials) was generated identically, but with the additional constraint that it had to be at least the set distance (controlled by the same distance parameter) from each of the other locations on that trial.

#### Staircasing

Both tasks were independently staircased to ensure that the tasks were difficult enough that participants had an incentive to attend selectively to the appropriate Location or Shape information. Each time a subject reported four correct answers on four successive trials of a task, the task-specific staircase parameter was adjusted to make the task more difficult. Each time a subject reported a single incorrect answer, the task-specific staircase parameter was adjusted to make the task easier. For the Location task, the staircased parameter was the *distance parameter* and was constrained to be within the range 0–4.24 degrees of visual angle. For the Shape task, the staircased parameter was the *distortion parameter* and was constrained to be within the range 0.07–1.06 degrees of visual angle.

#### fMRI procedure and preprocessing

fMRI data collection and preprocessing were identical to those of the Process experiment except as noted below. In the Format experiment, we collected 10.500 ± 1.033 runs (range 9–12), each containing 230 volumes (duration 460 s), from each subject. Functional data were registered to the structural scans from the Process experiment in order to place Format and Process data in a common space. Unlike for the Process experiment, for the Format experiment we performed motion correction using BrainVoyager's standard motion correction algorithm because there was no evidence that position or motion contributed to our decoding results in the Format experiment and because of the substantial time and resources that would be necessary for diffeomorphic motion correction.

#### Univariate fMRI analysis and functional region definition

Univariate analysis methods for the Format experiment were identical to those for the Process experiment except as noted below. In the Format experiment, for each of the Location and Shape trials, the set of event-related regressors included cue presentation epochs (1.8 s boxcar), encoding periods (2 s), delay periods (12 s), match-trial probes (duration of trial-based response time), and non-match-trial probes (duration of trial-based response time). We also included parallel regressors for error trial components, separately for each task and trial phase.

We identified functional ROIs via the conjunction of the open contrasts of the Location and Shape encoding period regressors (see Multivariate pattern analysis below for justification of the use of the encoding period). As in the Process experiment, prior to taking the conjunction of the open contrasts, each individual contrast map was smoothed with a Gaussian kernel (FWHM = 3 mm) to reduce any effects of noise and to account for minor variations in spatial alignment. GLM conjunction contrasts were thresholded at a voxelwise alpha of 0.05 and then corrected for multiple comparisons using the BrainVoyager Cluster-based Statistical Threshold Estimator plug-in to achieve a familywise error rate of 0.05, yielding an extent threshold of 93 functional voxels. As in the Process experiment, the resulting contrast maps were used to isolate regions of interest: local maximum contrast value voxels were identified in each activated brain region without constraints. Once the local maxima were identified, we created spherical ROIs (radius: 9 mm, including a total of 123 functional voxels) centered on these voxels. Following this procedure, in order to remove highly redundant ROIs, we culled ROIs that overlapped substantially with one another. The final set of Format ROIs is described in Supplementary Table S2.

#### Multivariate pattern analysis

Except as noted below, ROI-based and searchlight MVPA procedures for the Format experiment were identical to those for the Process experiment. In the Format experiment, we compared Location to Shape encoding periods in each Format ROI as well as each Process (see above), Modality (see below) and MD (Duncan, 2010) ROI. In effect, we tested whether neural activity underlying the encoding of visual items into internal perceptual or memory representations was sensitive to the Format demanded by the Location or Shape tasks. We focused on the encoding period rather than the later trial phases of the Format experiment for three reasons. First and foremost, we were interested in distinct Formats, regardless of whether information was in working memory or was represented using perceptual mechanisms; by using the encoding period, we were able to capitalize on both bottom-up sensory signals and top-down memory representation, increasing our sensitivity (see above). Second, we did not observe statistically significant decoding of Shape vs. Location during delay periods, an unsurprising result given that even such “easy” decoding problems as stimulus identity or orientation often show successful decoding only in visual (and sometimes portions of parietal) cortex. Third, the encoding period did not include motor responses, ruling out one nuisance source of decoding in motor regions of the brain during, for example, the probe/response period. In each ROI, we extracted peak decoding performance in the fMRI volumes 4–12 s after trial onset, which included the expected peak response after considering hemodynamic lag and the 2 s cue period prior to stimulus onset.

As in the Process experiment, we performed a searchlight MVPA analysis of the Format data using a time-compression procedure to capitalize on signal from the entire encoding period on each trial. After we subjected each individual task run to a fixed-effects, single-run GLM using regressors identical to those in the main GLM analysis described above, we extracted parameter weights for conditions of interest (Location encoding vs. Shape encoding) in each voxel in each locale and submitted these parameter estimates to searchlight MVPA.

### Sensorimotor modality experiment: experimental design and statistical analysis

The Modality experiment utilized 3-Tesla fMRI data originally collected for Tombu et al. (2011) and reanalyzed in Tamber-Rosenau et al. (2013). Briefly, the Modality experiment used 7–9 runs per subject with 20 slices of 4.5 mm thickness (0.5 mm gap), an in-plane resolution of 3.4375 × 3.4375 mm, a TR of 1.2 s, and approximately 301 s per run. On each trial of the Modality experiment, subjects performed either a visual-manual (VM) or an auditory-vocal (AV) stimulus-response mapping task (Figure 1C). The Modality experiment also included dual-task trials that are not analyzed here. To obtain Modality experiment ROIs (Supplementary Table S3), we used the ROIs originally defined in Tamber-Rosenau et al. (2013), which were obtained using a conjunction of open contrasts similar to that used for the Process and Format experiments. We do not perform ROI-based MVPA of the Modality experiment in the Modality (or MD) ROIs, as such an analysis has already been published (Tamber-Rosenau et al., 2013). However, for the present study we performed ROI-based MVPA of modality in the Process and Format ROIs, using identical procedures to the Process and Format experiments' ROI-based MVPA. In each ROI, we extracted peak decoding performance in the fMRI volumes 3.6–9.6 s after trial onset, which included the expected peak response after considering hemodynamic lag. Furthermore, not considering signal prior to 3.6 s ensures that we ignore any decoding that could have been driven by vocal artifact (which does not follow the conventional fMRI hemodynamic lag).

We also carried out a searchlight MVPA analysis of the Modality experiment. Prior to the searchlight MVPA analysis, we preprocessed the Modality experiment data similarly to the Process and Format data (for further details, see Tamber-Rosenau et al., 2013's Expt. 2), and then we regressed out motion estimates as well as z-transformed BOLD signals from four ventricle regions. These nuisance regressors were removed to account for signal artifacts due to the presence of vocal responses in the AV, but not VM, trials of the Modality data set. Taking this searchlight analysis of the Modality experiment together with those of the Process and Format experiments allowed us to examine the full space defined by all three hypothesized resource dimensions (see Introduction).

## Results

### Cognitive process experiment

#### Behavior

The Process experiment used a 6-alternative stimulus-response mapping task to load RS and a Go/No-Go (GNG) task to load RI (Figure 1A). Behavioral performance was high for both tasks (RS: accuracy = 92.09%, RT = 680 ms; RI: accuracy = 95.28%). As expected, in the RI task there was a significant reduction in performance [*t*_(15)_ = 9.1717, *p* = 1.5392 × 10^−7^] for No-Go trials (accuracy: 83.13%) compared to Go trials (accuracy: 97.71%), consistent with No-Go trials requiring inhibition of a prepotent Go response (e.g., Verbruggen and Logan, 2008). Consistent with the fact that RS demands, and thus response times, scale with choice complexity (Karlin and Kestenbaum, 1968; Van Selst and Jolicoeur, 1997; Marois et al., 2006), we observed slowed responses in the RS trials (680 ms) compared to Go trials (391 ms). Similarly, that the Go response was withheld on most No-Go trials suggests that we successfully evoked RI on No-Go trials.

#### Univariate fMRI analysis

**Figure 2:**
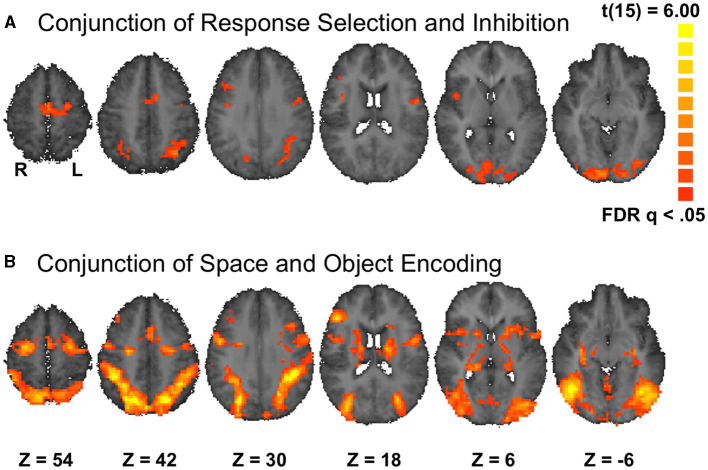
Functional ROIs. Results plotted on axial slices from the group-average Talairach-transformed brain. **(A)** Conjunction of open contrasts of No-Go and RS trials with matching stimuli, corrected for family-wise error to *p* = 0.05 using a cluster threshold of 42 functional voxels. **(B)** Conjunction of open contrasts of Location and Shape WM encoding, corrected for family-wise error to *p* = 0.05 using a cluster threshold of 93 functional voxels. Results of analyses within these ROIs are presented in Supplementary Figures S1–S4 and Supplementary Tables S1–S4.

The primary purpose of the univariate analysis was to identify overlapping activation for RS and RI, as expected of brain regions that contain a common neural resource for these two distinct processes. In turn, this overlap formed the basis of the Process ROIs in which we later applied MVPA. We observed overlap (Figure 2A, Supplementary Table S1) in regions broadly similar to the MD network, consistent with previous descriptions of task-invariant recruitment of MD regions (Duncan and Owen, 2000; Duncan, 2010; Fedorenko et al., 2013). Occipital visual cortex also survived the conjunction, presumably because both tasks relied on visual stimuli.

#### ROI multivariate timecourses

We evaluated whether each ROI contained neural ensembles that were specialized within the process dimension by using MVPA to decode between stimulus-matched RS and No-Go trials in a time-resolved fashion. All Process ROIs except a subset of visual cortex exhibited above-chance decoding of RS vs. RI (Supplementary Table S1). Process decoding argues against a single-resource model because the very brain regions that are recruited across multiple processes (based on univariate fMRI) distinguish between these processes, as revealed by spatial patterns of activation. Thus, MVPA yielded evidence against the process invariance expected of a single pluripotent resource. The major exception appeared to be in the occipital lobe, which is unsurprising given the identical visual stimuli.

### Representational format experiment

#### Behavior

The Format experiment used spatial location and object shape WM tasks to load location and shape representational formats, respectively (Figure 1B). Though staircasing kept task accuracies from both ceiling and floor (location: 80.08%, shape: 77.00%), accuracies [*t*_(15)_ = 2.9426, *p* = 0.0101] and response times [location: 1,374 ms, shape: 1,435 ms, *t*_(15)_ = −2.4495, *p* = 0.0271] differed across tasks. We focused our MVPA on the stimulus encoding period (approximately 13 seconds prior to responses), we only analyzed correct trials, and we mean-centered all MVPA analyses by removing region-based amplitude differences prior to MVPA [mean-centering limits contributions of difficulty or response time to decoding (Esterman et al., 2009; Tamber-Rosenau et al., 2011; Woolgar et al., 2014)]. Thus, these behavioral effects are unlikely to explain our MVPA results.

#### Univariate fMRI analysis

We identified overlapping activations for location and shape WM encoding, which then served as the basis for the Format ROIs. We focused on encoding because it produces more robust activation than maintenance of items in WM (see Materials & Methods). We observed overlap (Figure 2B, Supplementary Table S2) in regions that were broadly similar to the MD network and were more spatially extensive than the Process conjunction activity.

#### ROI multivariate timecourses

All Format ROIs exhibited above-chance decoding during the WM encoding period, except for regions of the right insula, subcortical and cerebellar regions, and some visual cortical regions (Supplementary Table S2). Based on the results of the Format experiment alone, it may be concluded that the right insula, in particular, could serve as a locus for a format-invariant resource, although with these results we cannot discard the possibility that portions of the visual cortex may also be format-invariant.

### Modality experiment

As the ROI-based modality results have been previously reported, we refer the reader to that publication's Table 3 (Tamber-Rosenau et al., 2013), where this experiment is discussed as Experiment 2. Briefly, all association cortex ROIs that were jointly activated across modalities in univariate analyses supported decoding of modality except for the DLPFC and anterior insula.

### Cross-experiment decoding of task dimensions

Both the Process and Format experiments revealed decoding of their respective dimensions in most cortical ROIs. Similarly, a previous analysis of Modality ROIs (Tamber-Rosenau et al., 2013) showed that most ROIs in that experiment supported decoding of modality (see that paper's Table 3). As explained in the Introduction, ROIs that support decoding of a task dimension do so because they contain specialized neural ensembles for that dimension, and these ROIs therefore do not embody single pluripotent neural populations. However, each experiment yielded some cortical ROIs that failed to decode that experiment's dimension, i.e., that might include neural ensembles that are invariant with respect to that experiment's dimension: anterior insula (AI) and dorsolateral prefrontal cortex (DLPFC) for Modality, visual cortical areas for Process, and areas of the insula and visual cortex for Format. The invariance of these ROIs to the dimension tested in their respective experiments makes them prime candidates to embody pluripotent flexible neural populations. However, any ROI that truly embodies such a population should be invariant to all theorized task dimensions, not just one of them. Thus, we subjected the ROIs isolated from each experiment (e.g., Format) to decoding within the two other dimensions (e.g., Process and Modality).

Most Process ROIs decoded format and modality (Supplementary Table S1). Most importantly, each Process ROI that failed to decode process (i.e., areas of visual cortex) successfully decoded both other dimensions. Similarly, most Format ROIs, including all Format ROIs that failed to decode format (i.e., areas of the insula and visual cortex), decoded process and modality (Supplementary Table S2). Finally, most Modality ROIs decoded process and format (Supplementary Table S3) and each Modality ROI that previously failed to decode modality (i.e., AI and DLPFC) successfully decoded at least one other dimension (Supplementary Figures S1, S2). Thus, every ROI contained neural ensembles that were specialized within at least one of the tested dimensions.

Because MD ROIs have been proposed to be domain-general flexible processors (Fedorenko et al., 2013), we repeated the MVPA in MD regions. Each MD ROI decoded along at least one dimension (Supplementary Figures S3, S4, Supplementary Table S4), suggesting that the domain-generality of MD activations stems from neighboring but distinct neural populations specialized for particular modalities, processes, or formats. Though decoding of task variables within MD regions is not novel (e.g., Woolgar et al., 2016), the present results extend this decoding to a broader range of task dimensions (see Introduction) and provide converging evidence that our task-based ROIs are comparable to previously-identified MD ROIs.

### Searchlight analysis of cognitive process, representational format, and sensorimotor modality

The ROI analysis provides only limited coverage of the brain, thus limiting conclusions about the cortical topography of decoding. Our ROI definition could have failed to detect some activation foci due to the conservative nature of conjunction analysis (Friston et al., 2005; Nichols et al., 2005), preventing the application of MVPA to additional relevant brain regions. Thus, we also performed whole-brain “searchlight” MVPA (Kriegeskorte et al., 2006). While the searchlight is able to explore the brain exhaustively, it uses smaller volumes of brain tissue for each pattern analysis, potentially reducing sensitivity. The searchlight analysis is also limited in its interpretative power in the case of brain regions that would be involved in only one task, as decoding in these regions could either mean that a single neural population is involved in one task and largely uninvolved in the other task, or that two neural populations are spatially segregated and differentially involved in the two tasks. Given these caveats, the searchlight analysis should be regarded as complementary to the ROI-based analysis.

**Figure 3:**
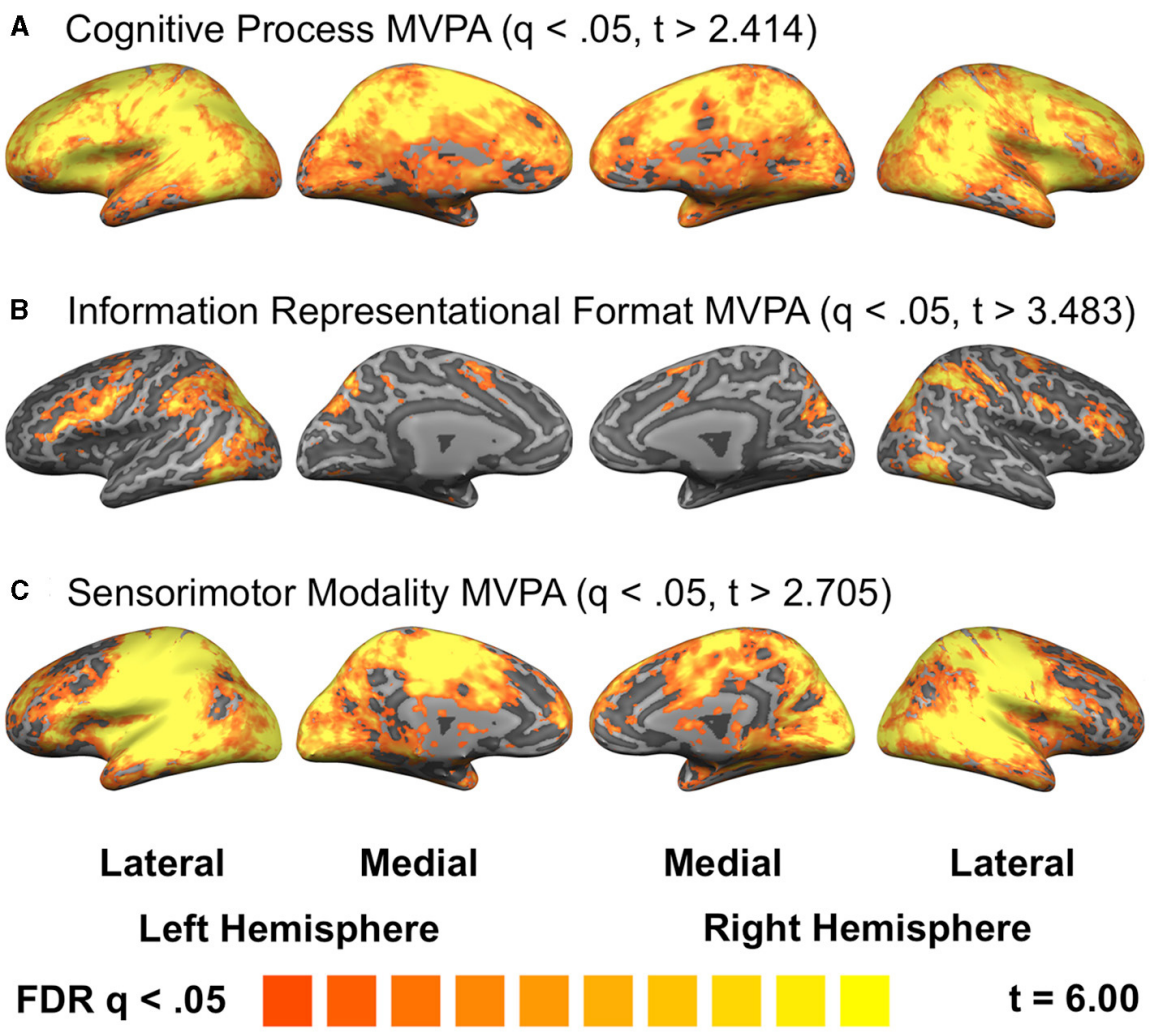
Searchlight decoding of individual dimensions. Results projected onto the inflated surface of the Talairach-transformed COLIN27 brain. Each experiment is thresholded at false discovery rate q < 0.05. **(A)** Process (response inhibition vs. response selection). **(B)** Format (location vs. shape encoding). **(C)** Modality (visual-manual vs. auditory-vocal response selection).

Decoding differed widely across task dimensions, both in extent—decoding of Process encompassed most of the cortical surface whereas decoding of Format was much more circumscribed—and topography. Topographic differences accorded with known functional specification: regions of early visual cortex failed to decode between identical visual stimuli in the Process experiment (Figure 3A; note absence of decoding near occipital pole consistent with centrally-presented visual stimulus); several frontal regions were invariant to modality (Figure 3C), consistent with prior univariate work (e.g., Dux et al., 2006; Ivanoff et al., 2009); and the Format experiment supported decoding mainly in dorsal and lateral frontal areas, parietal cortex, and ventral occipitotemporal areas (Figure 3B)—all thought to prefer either location or object-property formats such as shape (Mishkin et al., 1983; Goodale and Milner, 1992; Sala et al., 2003).

A notable result of the searchlight analysis was the extent of decoding of cognitive process (Figure 3A). Process decoding was observed in all four cortical lobes, and in fact in most of the cerebral cortex. This result is especially surprising because process decoding was exclusively performed between trials with identical stimulus conditions, subjected to the same data analysis, and thus could not have been driven by anything other than the process carried out in each of the two tasks. The lone difference between conditions aside from process was the presence of a button press for RS, but not No-Go, trials; this response difference is unlikely to have driven our results because we applied non-rigid-body motion correction, subsequently regressed out estimates of motion, and applied mean subtraction prior to MVPA (see Methods). Moreover, time-resolved ROI MVPA (Supplementary Figure S3) shows no evidence of motion-related signals, which would be expected to lead to spikes in decoding immediately after the response instead of following the canonical BOLD response delay.

The extensive process decoding we observed may not be that surprising, however, if information processing in the brain relies on the concerted efforts of many networks, resulting in widespread changes in brain states—coordinated by frontoparietal cortex (Cole et al., 2013)—across task conditions (Cole et al., 2014; Godwin et al., 2015). In accord with this view, performance of distinct cognitive tasks has been associated not only with traditional task-positive (Fox et al., 2005) or MD (Duncan and Owen, 2000; Duncan, 2010; Fedorenko et al., 2013) networks, but also with networks that were once thought to be related to rest, default, or “task-negative” states (Buckner et al., 2008; Andrews-Hanna et al., 2010; Passingham et al., 2010; Spreng et al., 2010, 2014; Gerlach et al., 2011; Leech et al., 2011; Mantini and Vanduffel, 2013) and with additional, topographically segregated, cortical networks (Dosenbach et al., 2006; Seeley et al., 2007; Vincent et al., 2008; Goulden et al., 2014). Consistent with this notion, a recent study has shown widespread, unselective recordings of task-relevant information across many regions of the mouse brain, spanning sensory cortex and much of the forebrain (Stringer et al., 2019). Taking these various findings together, much of cortex may be associated with task information processing, which could explain widespread process decoding. It is also possible, however, that large swathes of cortex may be involved exclusively with only one or the other process task, which would also lead to extremely widespread decoding in brain regions that did not pass the univariate conjunction analysis we used to define ROIs. Thus, we reiterate that significant decoding in this and other analyses should be used only to reject the hypothesis that the same neural populations or sub-regions are similarly engaged for the two conditions compared with MVPA.

**Figure 4:**
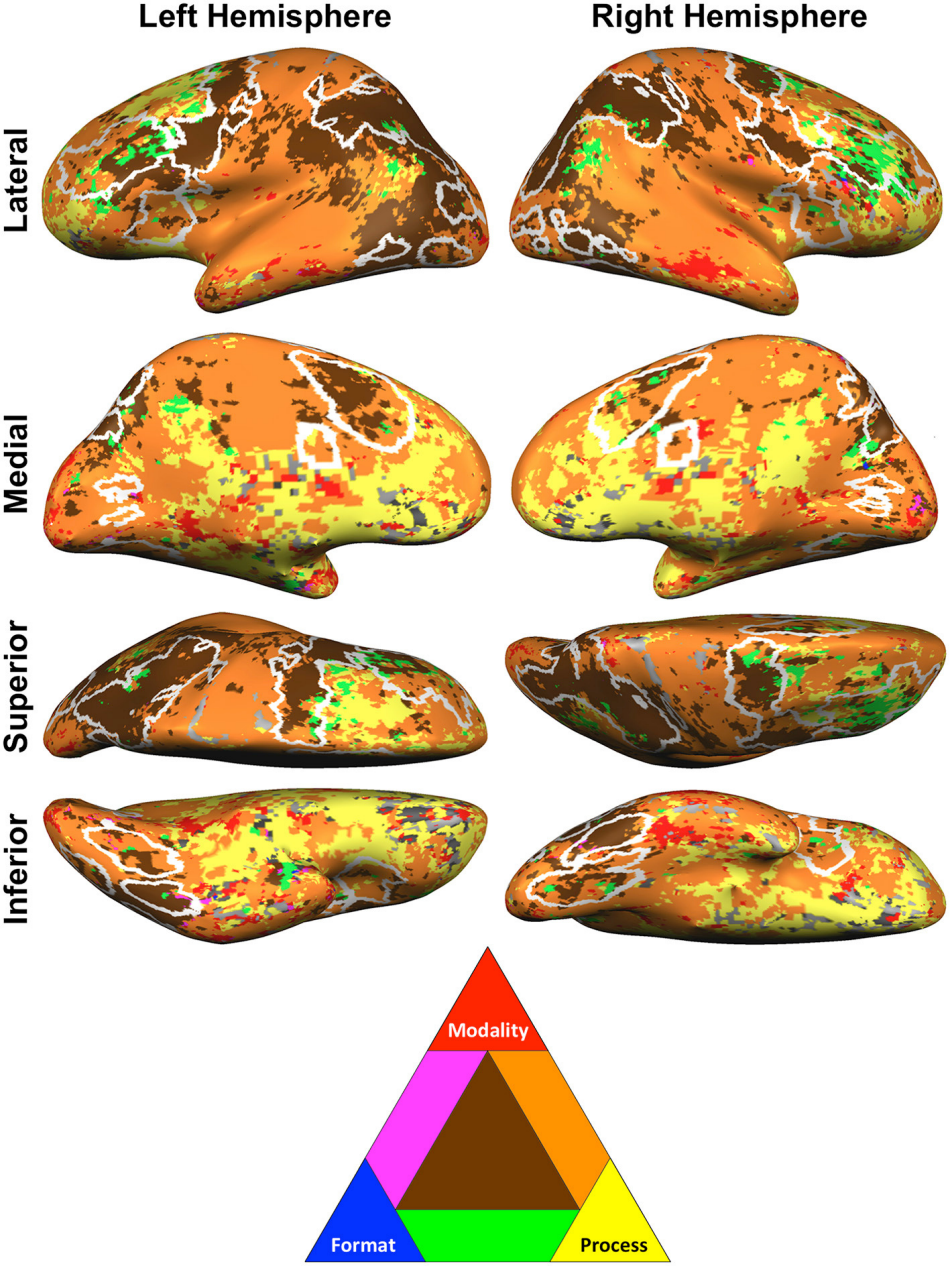
Overlap of searchlight decoding of process, format, and modality. Results projected onto the inflated surface of the Talairach-transformed COLIN27 brain. Each experiment is thresholded at false discovery rate q < 0.05. White outlines represent the MD network. We emphasize that the brown color represents areas of overlap of all dimensions of decoding; meaning that these locations had distinct decoding patterns (as detected via searchlight MVPA) for the different conditions of each task dimension: process, format, or modality. In other words, these regions contain the greatest heterogeneity of information decoding across the tested dimensions. These maps are best understood as complementary to those in Figure 3, which depict decoding for each individual dimension.

The widespread decoding patterns for each task dimensions notwithstanding, it is the analysis of the overlap of these patterns that is particularly enlightening. For one, this analysis revealed that decoding was observed for at least one task dimension in virtually all regions of cortex (Figure 4), ruling out the possibility that pluripotent neural ensembles underlie domain-general activation of any cortical area. The only notable exception to this result was orbitofrontal cortex near the rostral prefrontal MD ROIs, where proximity to sinus cavities reduces fMRI signal and requires special imaging sequences to compensate (e.g., Deichmann et al., 2003; Weiskopf et al., 2007). In support of this account, we observed no univariate conjunction activation, nor any single-task activation, within the rostral prefrontal MD ROIs except for an activation driven by the Location task of the Format experiment that partially overlapped the right rostral prefrontal MD ROI.

More importantly, the overlap analysis revealed that the regions that classify all three tested dimensions (Figure 4, brown) strongly overlapped with the MD network [Figure 4, white outlines, derived from Fedorenko et al. (2013), their Figure 1, and from http://imaging.mrc-cbu.cam.ac.uk/imaging/MDsystem]. Calculated on the cortical surface, 55.2% of the area subtended by regions decoding each of the three task dimensions overlapped with the MD network. Furthermore, most remaining cortex that decoded all three task dimensions is adjacent to MD cortex (compare Figure 4 brown to Figure 4 white outlines) and may correspond to the MD penumbra regions that show weaker co-activations (Assem et al., 2020). These findings are striking because they indicate recruitment of specialized neural populations in MD regions for distinct modalities, formats, and processes.

## Discussion

This research evaluated functional specification throughout the human cerebral cortex for pairs of tasks that varied along a multi-dimensional task space comprised of modalities, formats, and processes (c.f., Wickens, 1980, 2008). For each of these dimensions, we observed with univariate analyses several brain regions that were jointly engaged by two distinct tasks that varied along a single dimension. However, the multivariate analyses revealed that each of these cortical ROIs supported decoding of at least one task dimension, consistent with the notion that distinct functional domains—specialized sub-regions or neural ensembles (Kamitani and Tong, 2005, 2006; Peelen and Downing, 2007; Kamitani and Sawahata, 2010; Op de Beeck, 2010a,b; Lee and McCarthy, 2014)—can underlie univariate activation overlap across tasks. The same findings were obtained with searchlight MVPA; all cortical areas supported MVPA decoding of at least one task dimension. Together, these results constrain the extent of the flexibility of neural populations in the cerebral cortex to encode arbitrary categories within distinct task dimensions. Indeed, our results suggest that brain regions that are involved in a wide variety of tasks are mosaics of neural populations or sub-regions that are preferentially recruited for tasks varying along the dimensions of a classically-inspired matrix describing all cognitive tasks (c.f., Wickens, 1980, 1984, 2002, 2008). Thus, we suggest that even while these brain regions might be thought of as “domain general” at the scale of the larger ROI, they may be subdivided into spatially segregated neural populations or sub-regions that exhibit biases for particular modalities, processes, or formats. This is consistent with univariate findings of activation preferences for different task categories in association cortex (Assem et al., 2020, 2022, 2024).

The set of regions that classified all tested dimensions closely overlaps the MD network, which has been proposed to reflect flexible, domain-general cognition (Fedorenko et al., 2013). Our results suggest that functionally pluripotent neural populations equally capable to encode all three task dimensions may not underlie the ubiquitous MD activation observed in tasks that vary in modalities, processes, and formats because if identical populations encoded information without regard to changes along these task dimensions, spatial patterns of mean-centered fMRI signal in the MD network would not have allowed decoding between tasks (Tamber-Rosenau et al., 2013; Lee and McCarthy, 2014). Instead, adjacent or interleaved (e.g., Fedorenko et al., 2010, 2012; Michalka et al., 2015) functional domains or neural ensembles in the MD network underlie representations of distinct modalities, processes, and formats. The remarkable localization of decoding of all three multiple-resources dimensions to MD regions is likely to be meaningful because interleaved representations within a brain region facilitate functional interactions at similar scales of abstraction (e.g., Roe et al., 2009, 2012). Thus, even though our results found no evidence of functionally equipotent neural populations, they paradoxically reinforce the idea that MD regions serve multiple purposes because they are the primary cortical foci in which encoding of multiple task dimensions converges.

As noted above, previous studies have demonstrated decoding of task variables with MVPA in similar fronto-parietal MD regions to those we identified as supporting decoding of all task dimensions (see Woolgar et al., 2016 for review and meta-analysis). Our focus on a set of tasks with systematically varying modalities, formats, and processes addresses a distinct theoretical concept from these prior studies. Specifically, prior studies have focused on decoding sensory content within a single sensory modality, motor performance within a single motor modality, or task rule representations (e.g., specific rules that all shared a common if-then format) to assess whether specific representations are stored in these regions. These are important topics of investigation, but they investigate a narrower construct of flexibility compared to the present systematic study of encoding of task dimensions. In addition, our conceptualization of task dimensions provides ready integration between the present results and a decades-long psychology and human factors literature on information processing that validates the application of such conceptualization (e.g., Navon and Gopher, 1979; Wickens, 1980; Navon and Miller, 2002; Tombu and Jolicoeur, 2003; Boles et al., 2007; Salvucci and Taatgen, 2008; Proctor and Vu, 2010; Martins, 2016).

### Limitations and alternative explanations

Our results could be framed under a rapid adaptive coding framework—the idea that brain regions embodying flexibility contain pluripotent neural populations that represent immediately task-relevant information (Duncan, 2001, 2010). According to this framework, ensembles of neurons in a given flexible region may be initially functionally pluripotent but momentarily adopt a specific information code so that they represent a specific task dimension. However, to the degree that these codes are stable over the timescale of our experiments (i.e., hours)—a necessity for decoding, given the leave-one-run-out MVPA procedures—such codes are like any other population distinction within a broader brain region and constitute specialization of neural ensembles. Additionally, if rapid adaptive coding explained our results, such coding should be observed throughout the MD network because of the minimal functional distinction among MD regions (Woolgar et al., 2011; Erez and Duncan, 2015). However, we observed that MD regions are heterogeneous in the degree to which they represent different task variables, and that such heterogeneity generally reflects well-established organization of the cerebral cortex (e.g., modality-specific representations in posterior regions). Thus, we conclude that our results are unlikely to be solely explained by rapid adaptive coding within pluripotent neural populations. We do not mean to reject adaptive coding, as it is a likely mechanism by which arbitrary tasks can be encoded in a given cortical region. Rather, we suggest that adaptive coding is built on broad functional biases in neurons (analogous, for example, to orientation tuning in visual cortex) that lead to different neural populations representing distinct task dimensions for at least the duration of task performance.

The spatial resolution limitations of fMRI and MVPA prevent us from ruling out single pluripotent neurons. It is possible, for example, that two tasks varying along a given dimension recruit a common subset of neurons (see, e.g., Rao et al., 1997; Cromer et al., 2010) and that the pattern differences we observe using MVPA are driven by a small pool of neighboring but non-overlapping neurons recruited by each task. This account is unlikely, however, because MVPA is most sensitive to macroscopic patterns of neural populations on the scale of voxels (Op de Beeck, 2010a,b). Thus, if there are neurons that are truly pluripotent, they are most likely rare compared to those that show greater specificity.

We also cannot rule out the contribution of “mixed selectivity” to our signal. Mixed selectivity is the notion that neural populations can encode information that cannot be unambiguously extracted from single neurons' activity because each neuron's information content is highly dependent on the context of neighboring neurons' activity (Rigotti et al., 2013; Fusi et al., 2016). A similar consideration may apply at the scale of regions (vs. neural populations) and voxels (vs. single neurons or neural circuits). However, even if mixed selectivity is a property of the signals we report, it is not clear how it would relate to spatial decoding at the scale of voxels, especially since we removed ROI- or searchlight-wide BOLD amplitude differences. Thus, while mixed selectivity may exist, it does so orthogonally to our findings, which require spatial segregation of biases in large- (voxel-) scale neural populations or sub-regions to decode information.

Our decoding, even when significant, was not near 100%. First, we note that the decoding we observed is well within the range typically evaluated as meaningful in this literature; for example, significant decoding in similar brain regions in past studies is generally below 60% (e.g., Woolgar et al., 2011) and is often as low as 52% (e.g., Reverberi et al., 2011; Erez and Duncan, 2015). Moreover, there are a number of possible accounts to explain decoding levels well below 100%. For one, numerically low decoding may reflect the absence of model optimization (e.g., feature selection of voxels considered the most informative, c.f., Esterman et al., 2009; or, cost-parameter optimization, c.f. Tamber-Rosenau et al., 2011); studies that apply such optimizations often observe comparatively high MVPA performance (e.g., Kamitani and Tong, 2005; Polyn et al., 2005). Low decoding levels could also reflect the degree of spatial segregation of task-specific neural populations in each brain region; the more overlap between such neural populations the lesser the power to discriminate between their activity. That said, we note that our conclusions are primarily qualitative in nature; i.e., do we find significant evidence of segregation of function or not? Consistent with this viewpoint, meta-analysis of MVPA in the MD network has considered the localization of significant decoding, rather than its magnitude, as the yardstick for attributing specific task encoding to distinct brain regions (e.g., Woolgar et al., 2016).

Finally, while we focused on the cerebral cortex, we did not observe decoding of task dimensions in several subcortical areas. First, subcortical regions generally suffer from lower signal-to-noise ratio using standard fMRI imaging sequences, perhaps undermining our ability to decode in these regions. Second, the notion that MVPA mainly detects large-scale patterns (Op de Beeck, 2010a,b) may explain why several subcortical nuclei did not consistently support decoding in the present study: many subcortical structures are small and our methodology lacked the spatial resolution to distinguish separate neural populations in them because they contain too few functional voxels. Indeed, MVPA inherently requires the use of comparatively large volumes of tissue in each test because of the multi-voxel nature of the patterns it considers. ROIs at this scale most likely encompass multiple subcortical structures, reducing our sensitivity and specificity in these regions. Higher-resolution imaging could address these shortcomings in future studies. It is worth noting that the fact that subcortical regions do not support broad decoding of Process in particular provides further reassurance that the widespread decoding of this dimension cannot be attributed to, e.g., uncorrected motion or other volume-wide spurious causes.

## Conclusions

We examined tasks varying in three dimensions theorized by classic cognitive models and found that all regions of cortex contain spatially segregated neural populations that are functionally distinct for different categorical levels of at least one of these task dimensions. Thus, there are no uniformly functionally pluripotent neural ensembles in the human cerebral cortex that are measurable by MVPA applied to fMRI data. This finding constrains how brain regions with adaptive coding properties implement flexible cognition. In particular, these findings suggest that domain-general resources or bottlenecks postulated by classic psychological models are not likely to stem from truly domain-general neural resource pools. Paradoxically, these results also explain why a consistent cortical network underlies performance in a wide range of tasks (Fox et al., 2005; Duncan, 2010)—these are the very brain regions that contain closely juxtaposed neural ensembles specialized within all three task dimensions. We speculate that such proximity of specialized neural populations may facilitate neural interactions across these dimensions to efficiently encode task-relevant information in order to rapidly implement adaptive behavior.

## Data availability statement

The raw data supporting the conclusions of this article will be made available by the authors, without undue reservation.

## Ethics statement

The studies involving humans were approved by Vanderbilt University Institutional Review Board. The studies were conducted in accordance with the local legislation and institutional requirements. The participants provided their written informed consent to participate in this study.

## Author contributions

BT-R: Conceptualization, Formal analysis, Funding acquisition, Investigation, Methodology, Software, Writing – original draft, Writing – review & editing. AN: Investigation, Methodology, Writing – review & editing. RM: Conceptualization, Funding acquisition, Methodology, Resources, Supervision, Writing – review & editing.

## Footnotes

^1^A critique of the functional pluripotency account might be that a population that responds similarly to all tasks does not actually encode any information about the tasks. This is unlikely to be the case, as the ROI localizer analysis isolates regions that are robustly activated by each task prior to performing MVPA, all but ensuring that these regions encode some task-relevant information. The key point investigated here is whether the information that is encoded is stably segregated across neural ensembles biased toward processing each task (as would be detected by MVPA if the ROIs show functional specificity) or whether it is common across—or instantly adaptive to—the tasks (i.e., exhibits functional pluripotency), consistent with several classic psychological models that have proposed such a generalized “resource” for cognition (Moray, 1967; Kahneman, 1973; Tombu and Jolicoeur, 2003; Marois and Ivanoff, 2005; Tombu et al., 2011; Koch et al., 2018).
