# Supplementary material for: Flexible encoding of multiple task dimensions in human cerebral cortex
Source: Front Cognit. 2024 Jul 24;3:1438390. doi: 10.3389/fcogn.2024.1438390 (PMC13281067; doi:10.3389/fcogn.2024.1438390)
Supplement: Supplementary file 1 [file Data_Sheet_1.docx]

**Figure S1. Multivariate Pattern Analysis Timecourses from the Process Experiment in Modality-Invariant Regions**. Each plot represents decoding in one of the five modality-invariant regions of interest (ROIs) identified in Tamber-Rosenau et al. (2013). We observed above-chance decoding following the expected hemodynamic response shape in each ROI. Time 0 on the x-axis represents the onset of the stimulus disk. Heavy black line represents mean across participants. Each colored line represents an individual participant. Error bars represent one-tailed 95% confidence intervals, Bonferroni corrected for the 18 regions in the Modality ROI set.

**Figure S2. Multivariate Pattern Analysis Timecourses from the Format Experiment in Modality-Invariant Regions**. Each plot represents decoding in one of the five modality-invariant regions of interest (ROIs) identified in Tamber-Rosenau et al. (2013). We observed above-chance decoding at timepoints 8 and/or 10 s in each ROI, most likely reflecting the stimulus encoding period (i.e., 4 – 6 seconds of latency to peak decoding). Time 0 on the x-axis represents the onset of the task cue (cyan shading). The encoding period lasted from timepoints 2 – 4 s (red shading). The working memory delay lasted from 4 – 16 s (blue shading). Probe onset was at 16 s (green shading). Heavy black line represents mean across participants. Each colored line represents an individual participant. Error bars represent one-tailed 95% confidence intervals, Bonferroni corrected for the 18 regions in the modality ROI set.

**Figure S3. Multivariate Pattern Analysis Timecourses from the Process Experiment in Multiple Demand (MD) Regions**. Each plot represents decoding in one of the ten regions identified in Duncan (2010). We observed above-chance decoding following the expected hemodynamic response shape in each region except for Left Rostral PFC. Plotting convention is as in Figure S1. Error bars represent one-tailed 95% confidence intervals, Bonferroni corrected for the 10 MD regions. The numbers projected onto inflated cortical surfaces (extracted from a Talairach-transformed version of the COLIN27 brain) represent the locations of MD ROIs derived from Duncan (2010).

**Figure S4. Multivariate Pattern Analysis Timecourses from the Format Experiment in Multiple Demand (MD) Regions** Each plot represents decoding in one of the ten regions identified in Duncan (2010). We observed above-chance decoding at timepoints 8 and/or 10 s in each region except Right Rostral PFC, most likely reflecting the stimulus encoding period (i.e., 4 – 6 seconds of latency to peak decoding). Plotting convention is as in Figure S2. Error bars represent one-tailed 95% confidence intervals, Bonferroni corrected for the 10 MD regions.

**Table S1. Peak MVPA decoding performance in Process ROIs**.

|  | Talairach Coordinates | | | Peak MVPA in Cognitive Process Experiment | | | | Peak Encoding Period MVPA in Representational Format Experiment | | | | Peak MVPA in Modality Experiment | | | |
| --- | --- | --- | --- | --- | --- | --- | --- | --- | --- | --- | --- | --- | --- | --- | --- |
| Region | X | Y | Z | Time (s) | Accuracy (%) | t(15) | p | Time (s) | Accuracy (%) | t(15) | p | Time (s) | Accuracy (%) | t(11) | p |
| R Anterior Lateral PFC | 41 | 25 | 24 | ***4*** | ***56.70*** | ***5.6751*** | ***2.2032x10^-5^*** | ***8*** | ***62.03*** | ***6.4551*** | ***5.4231x10^-6^*** | ***6*** | ***62.14*** | ***4.4882*** | ***4.5947x10^-4^*** |
| R Lateral PFC | 32 | 13 | 33 | ***6*** | ***55.90*** | ***7.8414*** | ***5.5052x10^-7^*** | 8 | 57.53 | 2.5547 | 0.0110 | ***4.8*** | ***60.82*** | ***3.7527*** | ***0.0016*** |
| R Insula/Frontal Operculum | 41 | 4 | 6 | ***4*** | ***59.96*** | ***7.9905*** | ***4.3690x10^-7^*** | 10 | 57.50 | 3.2565 | 0.0027 | ***6*** | ***66.13*** | ***7.0213*** | ***1.1037x10^-5^*** |
| R Inferior Frontal Junction | 38 | -5 | 27 | ***4*** | ***58.11*** | ***5.8965*** | ***1.4674x10^-5^*** | ***8*** | ***63.07*** | ***4.5350*** | ***1.9740x10^-4^*** | ***6*** | ***70.72*** | ***8.9053*** | ***1.1623x10^-6^*** |
| R Anterior IPS | 29 | -50 | 39 | ***6*** | ***59.03*** | ***6.8634*** | ***2.6914x10^-6^*** | ***8*** | ***68.22*** | ***6.9687*** | ***2.2552x10^-6^*** | ***6*** | ***64.02*** | ***7.1132*** | ***9.7978x10^-6^*** |
| R Middle IPS | 32 | -56 | 39 | ***6*** | ***58.80*** | ***7.4049*** | ***1.1006x10^-6^*** | ***8*** | ***67.26*** | ***7.0913*** | ***1.8384x10^-6^*** | ***6*** | ***62.39*** | ***9.3323*** | ***7.3407x10^-7^*** |
| R Posterior IPS | 26 | -65 | 39 | ***6*** | ***58.84*** | ***7.9003*** | ***5.0228x10^-7^*** | ***8*** | ***69.73*** | ***9.7363*** | ***3.5470x10^-8^*** | ***7.2*** | ***64.90*** | ***5.5331*** | ***8.8603x10^-5^*** |
| R Medial Superior Parietal Lobule/Precuneus | 14 | -71 | 33 | ***4*** | ***59.33*** | ***8.0151*** | ***4.2068x10^-7^*** | ***8*** | ***64.40*** | ***5.8542*** | ***1.5851x10^-5^*** | ***4.8*** | ***63.70*** | ***5.5963*** | ***8.0606x10^-5^*** |
| R Anterior Calcarine Sulcus | 5 | -74 | 9 | 6 | 53.17 | 2.7914 | 0.0068 | ***8*** | ***56.38*** | ***4.6482*** | ***1.5769x10^-4^*** | ***6*** | ***63.07*** | ***5.1249*** | ***1.6547x10^-4^*** |
| R Ventral Visual Cortex | 29 | -83 | -12 | ***4*** | ***54.60*** | ***3.4699*** | ***0.0017*** | ***8*** | ***56.96*** | ***5.0355*** | ***7.3927x10^-5^*** | ***6*** | ***67.48*** | ***8.4782*** | ***1.8727x10^-6^*** |
| R Posterior Calcarine Sulcus | 11 | -89 | -6 | ***4*** | ***55.17*** | ***3.9131*** | ***6.9189x10^-4^*** | ***8*** | ***56.27*** | ***3.9317*** | ***6.6609x10^-4^*** | ***4.8*** | ***70.56*** | ***7.2402*** | ***8.3237x10^-6^*** |
| L Anterior Cingulate | -10 | 4 | 42 | ***4*** | ***60.36*** | ***5.5265*** | ***2.9052x10^-5^*** | ***8*** | ***60.30*** | ***7.7732*** | ***6.1243x10^-7^*** | ***7.2*** | ***61.88*** | ***5.0439*** | ***1.8784x10^-4^*** |
| L Inferior Frontal Junction | -43 | -8 | 33 | ***4*** | ***62.49*** | ***9.0291*** | ***9.4106x10^-8^*** | ***8*** | ***66.03*** | ***5.4984*** | ***3.0621x10^-5^*** | ***6*** | ***77.73*** | ***11.6863*** | ***7.6331x10^-8^*** |
| L Frontal Eye Field | -25 | -8 | 51 | ***4*** | ***61.23*** | ***7.6670*** | ***7.2394x10^-7^*** | ***8*** | ***68.84*** | ***10.1971*** | ***1.9314x10^-8^*** | ***6*** | ***61.73*** | ***6.4124*** | ***2.4973x10^-5^*** |
| L Superior Medial Frontal Cortex | -1 | -8 | 61 | ***4*** | ***63.74*** | ***9.1299*** | ***8.1612x10^-8^*** | ***8*** | ***64.34*** | ***5.1509*** | ***5.9188x10^-5^*** | ***6*** | ***71.70*** | ***10.1522*** | ***3.1780x10^-7^*** |
| L Inferior Parietal Lobule | -43 | -50 | 39 | ***4*** | ***58.95*** | ***8.6636*** | ***1.5922x10^-7^*** | ***8*** | ***67.19*** | ***6.7860*** | ***3.0683x10^-6^*** | ***6*** | ***70.26*** | ***6.3046*** | ***2.9002x10^-5^*** |
| L Anterior IPS | -34 | -53 | 33 | ***6*** | ***57.96*** | ***8.1636*** | ***3.3525x10^-7^*** | ***8*** | ***66.57*** | ***10.7024*** | ***1.0155x10^-8^*** | ***6*** | ***63.23*** | ***4.4698*** | ***4.7359x10^-4^*** |
| L Middle IPS | -31 | -59 | 39 | ***4*** | ***59.24*** | ***8.0106*** | ***4.2358x10^-7^*** | ***8*** | ***70.59*** | ***11.2864*** | ***4.9727x10^-9^*** | ***7.2*** | ***64.90*** | ***5.1405*** | ***1.6150x10^-4^*** |
| L Posterior IPS | -25 | -68 | 39 | ***6*** | ***57.92*** | ***5.6571*** | ***2.2778x10^-5^*** | ***8*** | ***70.14*** | ***11.9458*** | ***2.2994x10^-9^*** | ***6*** | ***65.71*** | ***5.9556*** | ***4.7579x10^-5^*** |
| L Ventral Visual Cortex | -34 | -77 | -6 | ***4*** | ***55.86*** | ***5.2627*** | ***4.7800x10^-5^*** | ***10*** | ***59.62*** | ***4.7193*** | ***1.3705x10^-4^*** | ***4.8*** | ***69.47*** | ***7.5128*** | ***5.9049x10^-6^*** |
| L Calcarine Sulcus | -7 | -86 | 3 | ***6*** | ***53.64*** | ***3.6047*** | ***0.0013*** | ***8*** | ***58.60*** | ***4.6875*** | ***1.4592x10^-4^*** | ***4.8*** | ***66.52*** | ***5.7746*** | ***6.1910x10^-5^*** |
| L Posterior Calcarine Sulcus | -19 | -89 | -6 | 4 | 53.29 | 2.5483 | 0.0111 | ***10*** | ***55.67*** | ***3.3587*** | ***0.0022*** | ***4.8*** | ***72.61*** | ***9.5107*** | ***6.0880x10^-7^*** |

Chance accuracy is 50%. All p-values reflect one-tailed tests, as the goal of each test was to ask if decoding exceeded chance. Tests are ***bolded and italicized*** when they are significant at alpha = 0.05, corrected for the 22 comparisons entailed by examining the 22 Process ROIs, equivalent to alpha = 0.0023. Peaks are defined as the maximum rate of decoding from the volumes 2 – 10 s after trial onset for the Process experiment, 4 – 12 s after trial onset for the Format experiment, and 3.6 – 9.6 s for the Modality experiment (see Methods).

**Table S2. Peak MVPA decoding performance in Format ROIs**.

|  | Talairach Coordinates | | | Peak MVPA in Cognitive Process Experiment | | | | Peak Encoding Period MVPA in Representational Format Experiment | | | | Peak MVPA in the Modality Experiment | | | |
| --- | --- | --- | --- | --- | --- | --- | --- | --- | --- | --- | --- | --- | --- | --- | --- |
| Region | X | Y | Z | Time (s) | Accuracy (%) | t(15) | p | Time (s) | Accuracy (%) | t(15) | p | Time (s) | Accuracy (%) | t(11) | p |
| R Dorsolateral PFC | 32 | 31 | 33 | ***6*** | ***56.87*** | ***5.3064*** | ***4.3991x10^-5^*** | ***10*** | ***60.87*** | ***4.9173*** | ***9.2988x10^-5^*** | 3.6 | 58.29 | 3.5979 | 0.0021 |
| R Ventrolateral PFC | 44 | 28 | 18 | ***4*** | ***55.72*** | ***3.9996*** | ***5.8012x10^-4^*** | ***8*** | ***64.25*** | ***7.6653*** | ***7.2587x10^-7^*** | ***3.6*** | ***62.94*** | ***5.7702*** | ***6.2309x10^-5^*** |
| R Insula/Frontal Operculum | 35 | 10 | 9 | ***4*** | ***61.05*** | ***8.9542*** | ***1.0468x10^-7^*** | 8 | 57.45 | 3.4643 | 0.0017 | ***6*** | ***62.44*** | ***5.2925*** | ***1.2766x10^-4^*** |
| R Frontal Operculum | 47 | 4 | 12 | ***4*** | ***58.15*** | ***7.5822*** | ***8.2823x10^-7^*** | ***8*** | ***61.16*** | ***4.4479*** | ***2.3483x10^-4^*** | ***6*** | ***67.46*** | ***6.8115*** | ***1.4545x10^-5^*** |
| R Inferior Frontal Junction | 47 | 1 | 24 | ***4*** | ***60.31*** | ***6.4333*** | ***5.6332x10^-6^*** | ***8*** | ***66.69*** | ***6.1071*** | ***1.0032x10^-5^*** | ***6*** | ***70.56*** | ***8.1365*** | ***2.7797x10^-6^*** |
| R Middle Insula | 35 | -5 | 9 | ***4*** | ***58.93*** | ***4.7961*** | ***1.1784x10^-4^*** | 8 | 57.20 | 3.4587 | 0.0018 | ***6*** | ***61.98*** | ***5.0145*** | ***1.9671x10^-4^*** |
| R Caudate | 14 | -5 | 15 | ***4*** | ***54.63*** | ***5.2569*** | ***4.8330x10^-5^*** | ***10*** | ***55.28*** | ***3.7386*** | ***9.8823x10^-4^*** | 3.6 | 61.91 | 3.9318 | 0.0012 |
| R Amygdala | 26 | -5 | -18 | 6 | 51.46 | 1.3778 | 0.0942 | 12 | 53.55 | 2.1242 | 0.0253 | ***3.6*** | ***61.88*** | ***3.9523*** | ***0.0011*** |
| R Frontal Eye Field | 23 | -11 | 45 | ***4*** | ***61.14*** | ***6.0802*** | ***1.0527x10^-5^*** | ***8*** | ***62.87*** | ***5.4911*** | ***3.1038x10^-5^*** | ***6*** | ***60.61*** | ***3.9974*** | ***0.0010*** |
| R Putamen | 26 | -11 | 15 | 4 | 54.46 | 3.4401 | 0.0018 | 4 | 53.06 | 2.0292 | 0.0303 | ***6*** | ***61.09*** | ***4.0536*** | ***9.5192x10^-4^*** |
| R Globus Pallidus | 17 | -11 | 3 | ***6*** | ***53.38*** | ***3.7736*** | ***9.1998x10^-4^*** | 4 | 53.16 | 2.3282 | 0.0171 | ***4.8*** | ***61.15*** | ***4.0165*** | ***0.0010*** |
| R Medial Temporal | 32 | -14 | -6 | ***6*** | ***54.16*** | ***4.5417*** | ***1.9480x10^-4^*** | 8 | 54.14 | 3.2807 | 0.0025 | ***6*** | ***63.93*** | ***7.9040*** | ***3.6629x10^-6^*** |
| R Pulvinar | 17 | -32 | 3 | ***6*** | ***53.36*** | ***3.9583*** | ***6.3095x10^-4^*** | 6 | 53.39 | 1.7865 | 0.0471 | ***3.6*** | ***66.39*** | ***6.3138*** | ***2.8631x10^-5^*** |
| R Inferior Parietal Lobule | 59 | -26 | 36 | ***4*** | ***57.56*** | ***5.7431*** | ***1.9434x10^-5^*** | ***8*** | ***62.01*** | ***6.2737*** | ***7.4572x10^-6^*** | ***6*** | ***73.10*** | ***6.6737*** | ***1.7489x10^-5^*** |
| R Anterior IPS | 26 | -50 | 36 | ***6*** | ***56.59*** | ***5.3416*** | ***4.1147x10^-5^*** | ***10*** | ***66.96*** | ***6.2140*** | ***8.2894x10^-6^*** | ***6*** | ***62.12*** | ***4.9331*** | ***2.2374x10^-4^*** |
| R Ventral Occipitotemporal | 35 | -50 | -9 | ***4*** | ***55.06*** | ***4.1863*** | ***3.9726x10^-4^*** | ***10*** | ***59.94*** | ***5.8462*** | ***1.6083x10^-5^*** | ***7.2*** | ***64.75*** | ***7.7538*** | ***4.3909x10^-6^*** |
| R Cerebellum | 2 | -53 | -9 | ***4*** | ***54.55*** | ***4.0070*** | ***5.7146x10^-4^*** | 10 | 53.79 | 2.6438 | 0.0092 | ***7.2*** | ***63.18*** | ***6.8171*** | ***1.4438x10^-5^*** |
| R Middle IPS | 26 | -65 | 36 | ***6*** | ***58.83*** | ***7.6545*** | ***7.3836x10^-7^*** | ***8*** | ***67.18*** | ***7.1739*** | ***1.6041x10^-6^*** | ***7.2*** | ***61.63*** | ***5.2813*** | ***1.2987x10^-4^*** |
| R Medial Superior Parietal Lobule/Precuneus | 5 | -68 | 48 | ***6*** | ***57.41*** | ***5.9714*** | ***1.2809x10^-5^*** | ***8*** | ***61.17*** | ***5.0013*** | ***7.8992x10^-5^*** | ***8.4*** | ***61.75*** | ***8.4676*** | ***1.8954x10^-6^*** |
| R Posterior IPS | 20 | -68 | 45 | ***6*** | ***57.40*** | ***5.0774*** | ***6.8183x10^-5^*** | ***8*** | ***65.34*** | ***6.4858*** | ***5.1402x10^-6^*** | ***6*** | ***69.95*** | ***7.0145*** | ***1.1135x10^-5^*** |
| R Ventral Occipital | 50 | -68 | -3 | ***6*** | ***55.53*** | ***4.8859*** | ***9.8848x10^-5^*** | ***8*** | ***59.49*** | ***5.0389*** | ***7.3449x10^-5^*** | ***6*** | ***65.16*** | ***4.7471*** | ***3.0123x10^-4^*** |
| R Anterior Calcarine | 11 | -74 | 6 | ***4*** | ***54.08*** | ***4.5896*** | ***1.7710x10^-4^*** | ***10*** | ***56.66*** | ***4.0084*** | ***5.6987x10^-4^*** | ***4.8*** | ***66.04*** | ***14.3079*** | ***9.3594x10^-9^*** |
| R Posterior Calcarine | 8 | -98 | 0 | 6 | 52.64 | 2.4713 | 0.0130 | ***8*** | ***56.55*** | ***4.5524*** | ***1.9068x10^-4^*** | ***4.8*** | ***68.52*** | ***6.7425*** | ***1.5945x10^-5^*** |
| L Lateral PFC | -37 | 19 | 30 | ***4*** | ***57.79*** | ***10.6329*** | ***1.1078x10^-8^*** | ***8*** | ***60.70*** | ***4.7947*** | ***1.1817x10^-4^*** | ***7.2*** | ***60.32*** | ***5.4076*** | ***1.0709x10^-4^*** |
| L Insula/Frontal Operculum | -34 | 7 | 6 | ***4*** | ***57.25*** | ***5.8627*** | ***1.5608x10^-5^*** | ***10*** | ***57.81*** | ***4.2539*** | ***3.4660x10^-4^*** | ***7.2*** | ***63.11*** | ***4.8647*** | ***2.4945x10^-4^*** |
| L Anterior Cingulate | -4 | 4 | 45 | ***4*** | ***60.51*** | ***5.1801*** | ***5.5970x10^-5^*** | ***8*** | ***64.20*** | ***7.1799*** | ***1.5884x10^-6^*** | ***7.2*** | ***66.02*** | ***5.5737*** | ***8.3367x10^-5^*** |
| L Frontal Operculum | -46 | 1 | 12 | ***4*** | ***58.20*** | ***5.0264*** | ***7.5243x10^-5^*** | ***10*** | ***59.26*** | ***4.0812*** | ***4.9147x10^-4^*** | ***6*** | ***71.09*** | ***6.6881*** | ***1.7153x10^-5^*** |
| L Inferior Frontal Junction | -46 | -2 | 24 | ***4*** | ***58.60*** | ***8.0227*** | ***4.1578x10^-7^*** | ***8*** | ***66.60*** | ***5.4496*** | ***3.3555x10^-5^*** | ***6*** | ***72.46*** | ***10.3357*** | ***2.6550x10^-7^*** |
| L Superior Medial Frontal Cortex | -1 | -5 | 54 | ***4*** | ***62.59*** | ***7.2564*** | ***1.4010x10^-6^*** | ***8*** | ***64.41*** | ***5.5393*** | ***2.8361x10^-5^*** | ***7.2*** | ***70.46*** | ***8.2182*** | ***2.5265x10^-6^*** |
| L Caudate | -16 | -8 | 18 | 6 | 52.97 | 2.2675 | 0.0193 | ***8*** | ***56.07*** | ***4.3673*** | ***2.7597x10^-4^*** | 6 | 57.32 | 3.0556 | 0.0055 |
| L Frontal Eye Field | -28 | -11 | 48 | ***4*** | ***62.99*** | ***9.2936*** | ***6.4922x10^-8^*** | ***8*** | ***67.03*** | ***6.7731*** | ***3.1366x10^-6^*** | ***6*** | ***67.17*** | ***9.4639*** | ***6.3923x10^-7^*** |
| L Globus Pallidus | -16 | -11 | 0 | 4 | 53.57 | 3.1382 | 0.0034 | 6 | 54.00 | 3.3607 | 0.0021 | ***3.6*** | ***60.65*** | ***4.6665*** | ***3.4322x10^-4^*** |
| L Putamen | -25 | -14 | 18 | 6 | 53.18 | 3.1989 | 0.0030 | 12 | 53.88 | 1.9367 | 0.0359 | 3.6 | 57.35 | 3.0252 | 0.0058 |
| L Medial Temporal | -37 | -17 | -12 | ***6*** | ***53.95*** | ***4.7421*** | ***1.3103x10^-4^*** | 10 | 55.35 | 2.2143 | 0.0214 | ***6*** | ***66.11*** | ***5.2218*** | ***1.4236x10^-4^*** |
| L Inferior Parietal Lobule | -52 | -29 | 33 | ***4*** | ***62.08*** | ***8.0268*** | ***4.1316x10^-7^*** | ***10*** | ***65.95*** | ***7.0276*** | ***2.0438x10^-6^*** | ***6*** | ***88.80*** | ***17.8964*** | ***8.7726x10^-10^*** |
| L Pulvinar | -19 | -35 | 3 | 4 | 54.10 | 3.4377 | 0.0018 | 8 | 54.75 | 2.4332 | 0.0140 | 3.6 | 58.41 | 3.8929 | 0.0013 |
| L Anterior IPS | -37 | -41 | 36 | ***4*** | ***59.98*** | ***6.9455*** | ***2.3444x10^-6^*** | ***10*** | ***68.02*** | ***9.0665*** | ***8.9255x10^-8^*** | ***6*** | ***75.96*** | ***8.0320*** | ***3.1443x10^-6^*** |
| L Middle IPS | -28 | -53 | 36 | ***4*** | ***57.59*** | ***5.7701*** | ***1.8492x10^-5^*** | ***8*** | ***67.61*** | ***8.9567*** | ***1.0431x10^-7^*** | ***6*** | ***63.39*** | ***6.6239*** | ***1.8703x10^-5^*** |
| L Ventral Occipitotemporal | -37 | -62 | -6 | ***6*** | ***55.97*** | ***7.2792*** | ***1.3498x10^-6^*** | ***10*** | ***65.51*** | ***7.1333*** | ***1.7152x10^-6^*** | ***6*** | ***68.40*** | ***7.3395*** | ***7.3383x10^-6^*** |
| L Lingual Gyrus | -1 | -68 | 3 | 4 | 54.86 | 3.5887 | 0.0013 | ***10*** | ***55.54*** | ***4.8478*** | ***1.0650x10^-4^*** | ***7.2*** | ***61.50*** | ***4.2534*** | ***6.7895x10^-4^*** |
| L Posterior IPS | -19 | -71 | 45 | ***6*** | ***56.86*** | ***5.8998*** | ***1.4586x10^-5^*** | ***10*** | ***65.07*** | ***7.9677*** | ***4.5253x10^-7^*** | ***8.4*** | ***65.15*** | ***5.2732*** | ***1.3152x10^-4^*** |
| L Ventral Occipital | -46 | -74 | -9 | ***4*** | ***55.96*** | ***4.7665*** | ***1.2488x10^-4^*** | ***10*** | ***60.88*** | ***4.7552*** | ***1.2770x10^-4^*** | ***6*** | ***66.86*** | ***6.3334*** | ***2.7861x10^-5^*** |
| L Posterior Calcarine | -19 | -92 | 3 | ***6*** | ***53.70*** | ***4.0128*** | ***5.6418x10^-4^*** | ***8*** | ***57.96*** | ***8.0272*** | ***4.1290x10^-7^*** | ***4.8*** | ***73.39*** | ***10.9471*** | ***1.4860x10^-7^*** |

Chance accuracy is 50%. All p-values reflect one-tailed tests, as the goal of each test was to ask if decoding exceeded chance. Tests are ***bolded and italicized*** when they are significant at alpha = 0.05, corrected for the 43 comparisons entailed by examining the 43 Representational Format-defined ROIs, equivalent to alpha = 0.0012. Peaks are defined as the maximum rate of decoding from the volumes 2 – 10 s after trial onset for the Process experiment, 4 – 12 s after trial onset for the Format experiment, and 3.6 – 9.6 s for the Modality experiment (see Methods).

**Table S3. Peak MVPA decoding performance in Modality ROIs**.

|  | Talairach Coordinates | | | Peak MVPA in Cognitive Process Experiment | | | | Peak Encoding Period MVPA in Representational Format Experiment | | | |
| --- | --- | --- | --- | --- | --- | --- | --- | --- | --- | --- | --- |
| Region | X | Y | Z | Time (s) | Accuracy (%) | t(15) | p | Time (s) | Accuracy (%) | t(15) | p |
| R DLPFC | 29 | 31 | 33 | ***6*** | ***57.05*** | ***5.7254*** | ***2.0078x10^-5^*** | ***10*** | ***60.37*** | ***6.1201*** | ***9.8011x10^-6^*** |
| R pLPFC | 44 | 10 | 30 | ***4*** | ***58.68*** | ***6.6343*** | ***3.9763x10^-6^*** | ***8*** | ***61.57*** | ***3.9030*** | ***7.0624x10^-4^*** |
| R Anterior Insula | 32 | 7 | 9 | ***4*** | ***59.65*** | ***9.0779*** | ***8.7828x10^-8^*** | ***8*** | ***57.81*** | ***4.6817*** | ***1.4759x10^-4^*** |
| R Frontal Eye Field | 26 | -14 | 51 | ***4*** | ***61.00*** | ***6.1560*** | ***9.1914x10^-6^*** | ***8*** | ***65.16*** | ***5.0055*** | ***7.8348x10^-5^*** |
| R Temporoparietal Junction | 44 | -47 | 18 | ***4*** | ***57.41*** | ***6.4618*** | ***5.3594x10^-6^*** | ***8*** | ***58.93*** | ***6.0739*** | ***1.0647x10^-5^*** |
| R IPS | 23 | -68 | 45 | ***6*** | ***57.44*** | ***5.1392*** | ***6.0541x10^-5^*** | ***8*** | ***64.86*** | ***6.0592*** | ***1.0932x10^-5^*** |
| M Anterior Cingulate | 0 | 7 | 38 | ***4*** | ***62.04*** | ***8.4852*** | ***2.0694x10^-7^*** | ***10*** | ***61.26*** | ***5.4948*** | ***3.0824x10^-5^*** |
| M Posterior Anterior Cingulate | 0 | 4 | 45 | ***4*** | ***61.77*** | ***6.3351*** | ***6.6918x10^-6^*** | ***8*** | ***65.01*** | ***6.9944*** | ***2.1601x10^-6^*** |
| M Sensorimotor Area | 0 | -8 | 66 | ***6*** | ***57.28*** | ***4.9495*** | ***8.7341x10^-5^*** | ***8*** | ***59.73*** | ***5.0812*** | ***6.7680x10^-5^*** |
| M Medial Superior Parietal Lobule | 0 | -56 | 48 | ***4*** | ***57.37*** | ***4.9162*** | ***9.3181x10^-5^*** | ***10*** | ***61.21*** | ***5.3255*** | ***4.2424x10^-5^*** |
| L DLPFC | -28 | 40 | 33 | ***6*** | ***57.94*** | ***7.2889*** | ***1.3287x10^-6^*** | ***8*** | ***56.78*** | ***3.4563*** | ***0.0018*** |
| L Posterior DLPFC | -40 | 19 | 33 | ***4*** | ***57.27*** | ***8.5942*** | ***1.7623x10^-7^*** | ***10*** | ***61.00*** | ***6.6784*** | ***3.6865x10^-6^*** |
| L pLPFC | -49 | 4 | 36 | ***4*** | ***57.44*** | ***6.9506*** | ***2.3243x10^-6^*** | ***8*** | ***66.97*** | ***10.5147*** | ***1.2858x10^-8^*** |
| Left Anterior Insula | -30 | 10 | 12 | ***4*** | ***55.96*** | ***7.7997*** | ***5.8748x10^-7^*** | ***8*** | ***58.46*** | ***5.2203*** | ***5.1829x10^-5^*** |
| L Frontal Eye Field | -28 | -14 | 48 | ***4*** | ***62.91*** | ***11.6680*** | ***3.1684x10^-9^*** | ***8*** | ***66.36*** | ***6.6946*** | ***3.5857x10^-6^*** |
| L Lateral Frontal Eye Field | -37 | -8 | 51 | ***4*** | ***60.77*** | ***7.1986*** | ***1.5402x10^-6^*** | ***8*** | ***64.65*** | ***5.7889*** | ***1.7865x10^-5^*** |
| L SPL | -37 | -50 | 42 | ***4*** | ***60.69*** | ***9.1137*** | ***8.3503x10^-8^*** | ***8*** | ***68.43*** | ***7.1312*** | ***1.7212x10^-6^*** |
| L IPS | -25 | -71 | 42 | ***6*** | ***56.35*** | ***5.3313*** | ***4.1955x10^-5^*** | ***8*** | ***66.24*** | ***8.8220*** | ***1.2653x10^-7^*** |

Chance accuracy is 50%. All p-values reflect one-tailed tests, as the goal of each test was to ask if decoding exceeded chance. Tests are ***bolded and italicized*** when they are significant at alpha = 0.05, corrected for the 18 comparisons entailed by examining the 18 Modality ROIs, equivalent to alpha = 0.0028. Peaks are defined as the maximum rate of decoding from the volumes 2 – 10 s after trial onset for the Process experiment and 4 – 12 s after trial onset for the Format experiment (see Methods). We do not include decoding results for the Modality experiment here because ROI analyses of the Modality experiment in Modality ROIs have previously been published (Tamber-Rosenau et al., 2013).

**Table S4. Peak MVPA decoding performance in Multiple Demand (MD) ROIs**.

|  | Talairach Coordinates | | | Peak MVPA in Cognitive Process Experiment | | | | Peak Encoding Period MVPA in Representational Format Experiment | | | |
| --- | --- | --- | --- | --- | --- | --- | --- | --- | --- | --- | --- |
| Region | X | Y | Z | Time (s) | Accuracy (%) | t(15) | p | Time (s) | Accuracy (%) | t(15) | p |
| R Rostral PFC | 20 | 38 | -8 | ***4*** | ***53.53*** | ***3.3678*** | ***0.0021*** | 12 | 54.83 | 2.4980 | 0.0123 |
| R Inferior Frontal Sulcus | 40 | 23 | 29 | ***4*** | ***56.40*** | ***6.1246*** | ***9.7224x10^-6^*** | ***8*** | ***61.73*** | ***5.6941*** | ***2.1270x10^-5^*** |
| R Anterior Insula/Frontal Operculum | 33 | 15 | 5 | ***4*** | ***59.76*** | ***6.5851*** | ***4.3280x10^-6^*** | ***8*** | ***58.63*** | ***4.6296*** | ***1.6362x10^-4^*** |
| R IPS | 38 | -53 | 38 | ***6*** | ***60.11*** | ***8.9858*** | ***1.0007x10^-7^*** | ***8*** | ***65.59*** | ***8.3452*** | ***2.5493x10^-7^*** |
| M Anterior Cingulate | 0 | 30 | 22 | ***6*** | ***55.72*** | ***5.8252*** | ***1.6716x10^-5^*** | ***8*** | ***56.64*** | ***5.1793*** | ***5.6059x10^-5^*** |
| M PreSMA | 0 | 20 | 45 | ***4*** | ***57.60*** | ***6.8204*** | ***2.8946x10^-6^*** | ***8*** | ***59.76*** | ***5.2290*** | ***5.0970x10^-5^*** |
| L Rostral PFC | -21 | 38 | -9 | 6 | 52.91 | 2.1656 | 0.0234 | ***8*** | ***55.14*** | ***3.6700*** | ***0.0011*** |
| L Inferior Frontal Sulcus | -40 | 22 | 28 | ***6*** | ***56.55*** | ***5.6397*** | ***2.3524x10^-5^*** | ***8*** | ***64.98*** | ***7.4006*** | ***1.1082x10^-6^*** |
| L Anterior Insula/Frontal Operculum | -34 | 15 | 4 | ***4*** | ***59.54*** | ***10.0627*** | ***2.3009x10^-8^*** | ***10*** | ***59.21*** | ***4.9191*** | ***9.2651x10^-5^*** |
| L IPS | -37 | -53 | 39 | ***4*** | ***59.79*** | ***10.9748*** | ***7.2515x10^-9^*** | ***8*** | ***68.57*** | ***8.3867*** | ***2.3958x10^-7^*** |

Chance accuracy is 50%. All p-values reflect one-tailed tests, as the goal of each test was to ask if decoding exceeded chance. Tests are ***bolded and italicized*** when they are significant at alpha=0.05, corrected for the 10 comparisons entailed by examining the 10 MD ROIs, equivalent to alpha = 0.005. Peaks are defined as the maximum rate of decoding from the volumes 2 – 10 s after trial onset for the Process experiment and 4 – 12 s after trial onset for the Format experiment (see Methods). We do not include decoding results for the Modality experiment here because analyses of the Modality experiment in MD ROIs have previously been published (Tamber-Rosenau et al., 2013).

**References**

Duncan J (2010) The multiple-demand (MD) system of the primate brain: mental programs for intelligent behaviour. Trends Cogn Sci 14:172-179.

Tamber-Rosenau BJ, Dux PE, Tombu MN, Asplund CL, Marois R (2013) Amodal processing in human prefrontal cortex. The Journal of neuroscience : the official journal of the Society for Neuroscience 33:11573-11587.
