# Supplementary figures and images for: Flexible encoding of multiple task dimensions in human cerebral cortex
Source: Front Cognit. 2024 Jul 24;3:1438390. doi: 10.3389/fcogn.2024.1438390 (PMC13281067; doi:10.3389/fcogn.2024.1438390)

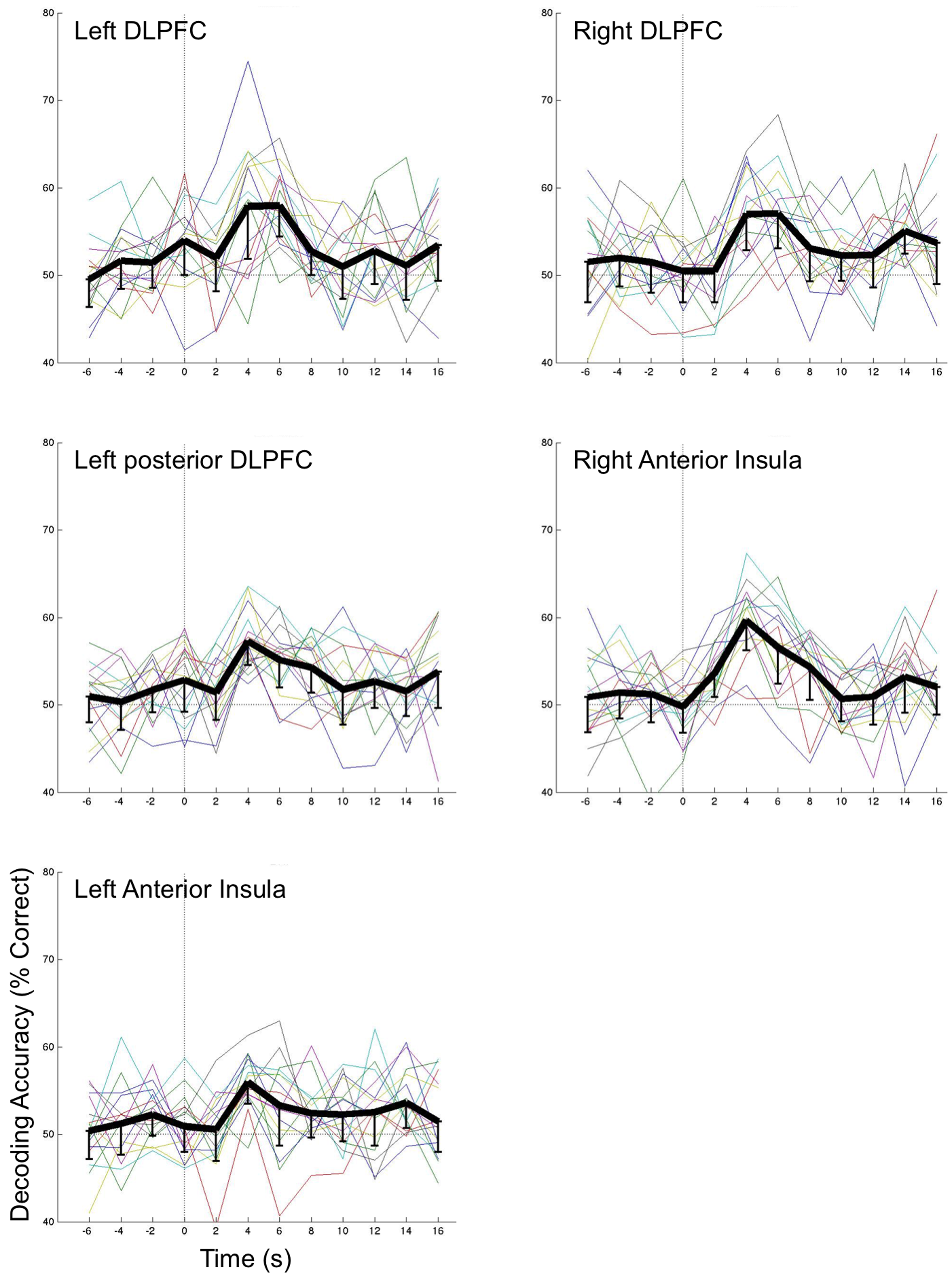

Supplement: Supplementary file 2 [file Image_1.TIF]

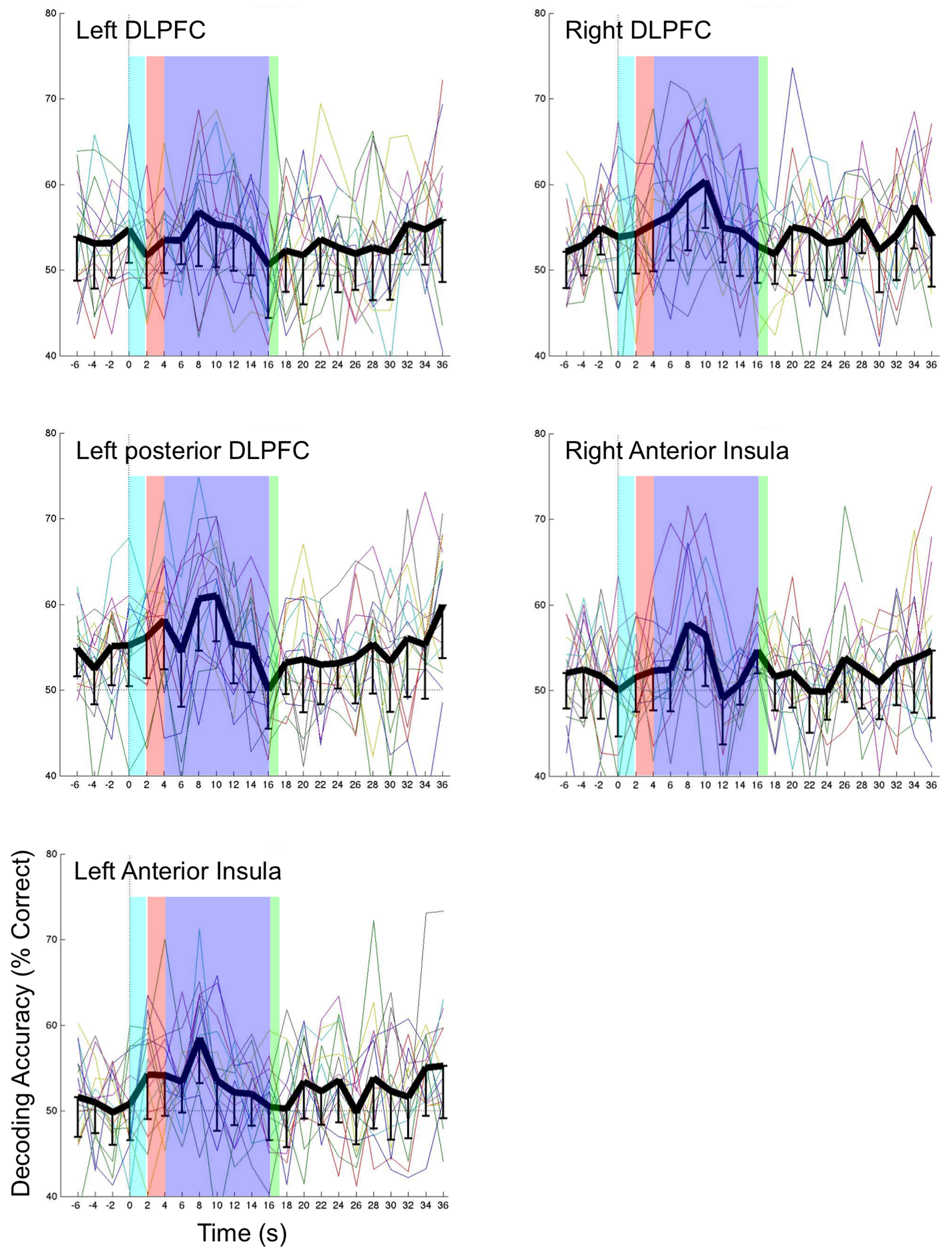

Supplement: Supplementary file 3 [file Image_2.TIF]

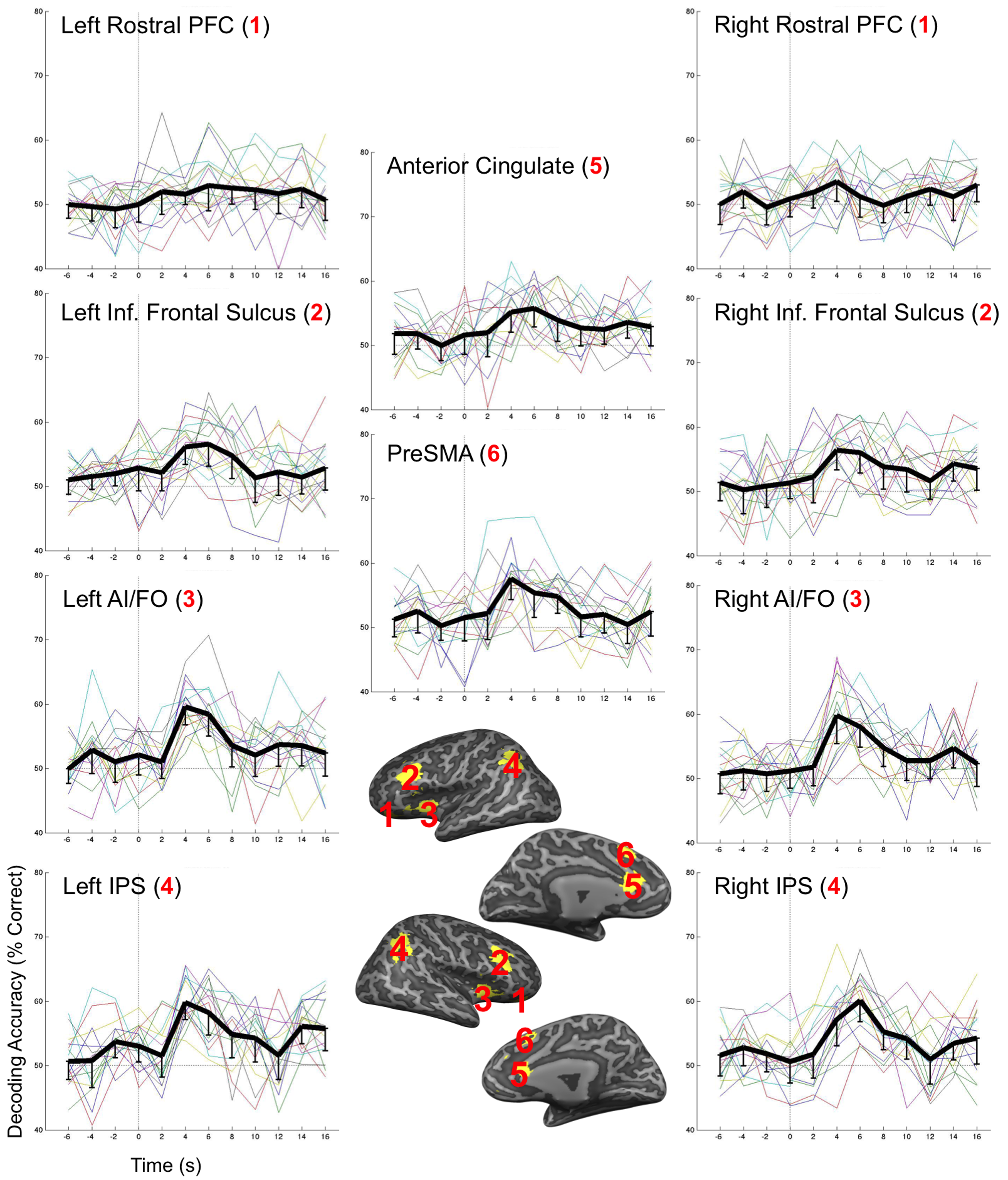

Supplement: Supplementary file 4 [file Image_3.TIF]

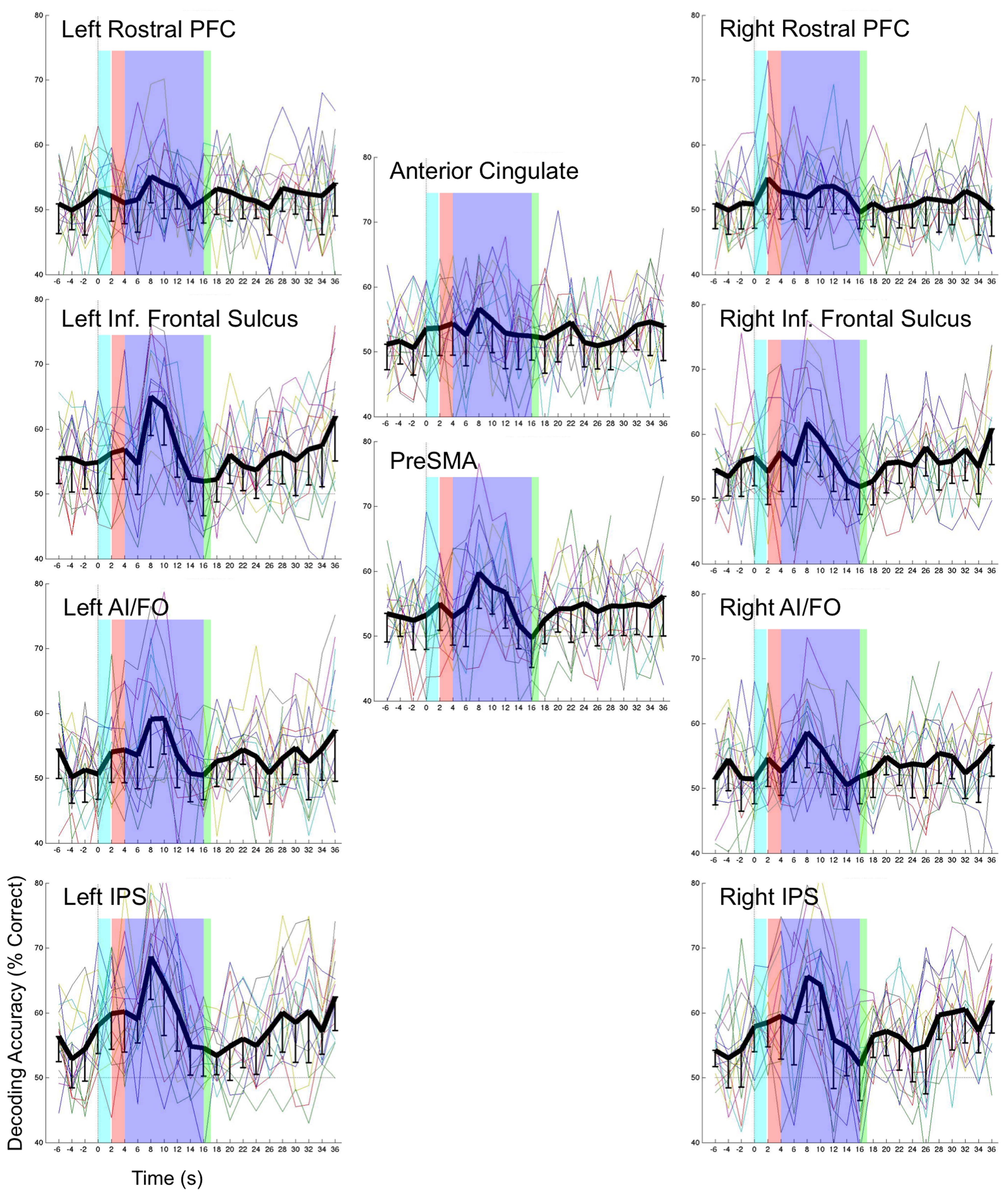

Supplement: Supplementary file 5 [file Image_4.TIF]
